# Nitroarene-Mediated Photo-oxidative Deconstruction and Upcycling of Unsaturated Rubber Waste

**DOI:** 10.1021/acs.macromol.5c02306

**Published:** 2026-01-22

**Authors:** Mengdong Guo, Zefeng Wang, Roberto Obregon, Obed Fernando, Junpeng Wang

**Affiliations:** School of Polymer Science and Polymer Engineering, 1687 The University of Akron, 170 University Ave, Akron, Ohio 44325, United States

## Abstract

Transforming waste polymers into materials with improved properties offers a compelling strategy for advancing the sustainable use of polymers, echoing the Chinese proverb: “Blue comes from indigo but surpasses it in blueness.” Herein, we report a photo-oxidative deconstruction and upcycling method for unsaturated rubber waste. Under 390 nm LED irradiation at room temperature, in the presence of a nitroarene oxidant, diverse unsaturated polymers—including natural rubber, polybutadiene, nitrile rubber, styrene–butadiene rubber, and even cross-linked nitrile gloves—are selectively converted into carbonyl-terminated oligomers. The aldehyde-functionalized products are cross-linked via dynamic imine chemistry with *p*-phenylenediamine to yield reprocessable elastomers with markedly improved tensile strength (from 12.9 to 16.7 MPa) and surface hydrophobicity (contact angle from 23 ± 2° to 83 ± 3°). Furthermore, the aldehyde groups on the oligomers can be selectively oxidized to carboxylic acids or reduced to hydroxyl groups, enabling their versatile use as polymeric additives including curing agents, chain extenders, and toughening agents. This strategy demonstrates a versatile route for converting rubber waste into high-value functional materials.

## Graphical abstract

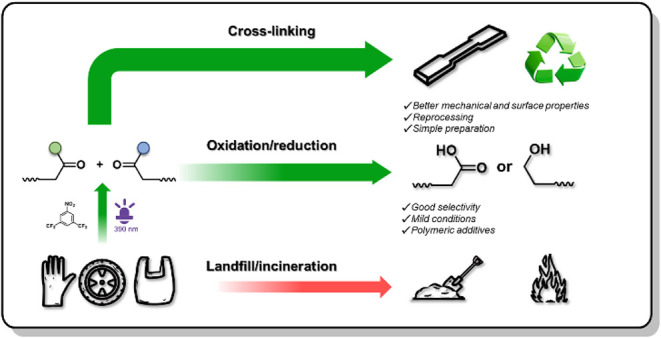

## Introduction

Rubber poses a major global challenge at the end of its life, with annual consumption reaching 31.5 million metric tons in 2024 due to its ubiquitous use in tires, elastomers, and disposable gloves.^1^ Most waste rubber is currently disposed of via landfilling or incineration, causing significant environmental and economic impacts due to the release of toxic byproducts such as benzene, dioxins, and furans.^2^ Recycling of rubber waste faces substantial hurdles compounded by (1) the structural complexity of unsaturated polymers, where diverse olefin configurations (cis, trans, vinyl) impede reprocessing; (2) the generation of harmful byproducts when recycling polydienes containing chlorine or other heteroatoms; (3) the energy-intensive nature of thermal depolymerization, which often yields mixed, low-value products; (4) difficulties in purification and repolymerization due to additives such as fillers and flame retardants; and (5) chemical inertness of cross-linked networks formed during vulcanization, which resist conventional recycling methods.^3–5^ Addressing these challenges is critical not only for obtaining valuable degradation products but also for transforming waste polymers into materials with enhanced mechanical performance and environmental compatibility.^6^

Current rubber deconstruction methods primarily involve metathesis, oxidation, and backbone rearrangement.^2,7–9^ Among these methods, oxidative cleavage offers distinct advantages: operational simplicity, high efficiency, and reliance on commercial oxidants without expensive catalysts.^10^ Conventional alkene oxidative cleavage methods—such as ozonolysis, permanganate oxidation, or peroxybenzoic acid oxidation—effectively degrade unsaturated polymers into oligomers and small molecules.^11,12^ However, these approaches have limitations; peroxybenzoic acid oxidation, for example, requires multiple strong oxidants and strict temperature control (0 °C) and poses a risk of epoxidation.^13,14^ In 2024, our group developed a photomediated oxidative degradation process using an earth-abundant Mn catalyst under O_2_. While effective, this method produced low acetal yields, required complex reaction components and a specialized metal catalyst, and exhibited solvent sensitivity. Consequently, the development of general, mild, and highly selective photochemical methods for waste rubber depolymerization is critically needed. Nitroarene serves as a potent reagent for alkene cleavage and diol construction at the molecular level.^15–17^ It has been successfully employed in the selective photooxidation of commodity postconsumer polyolefins to generate polymers featuring in-chain ketones.^18^

Recently, the Sumerlin group reported the photo-oxidative degradation of olefinic polymers using nitroarene as the oxidant.^19^ To advance this approach toward practical circularity, our research focuses on converting degradation products into high-value materials. We first optimized the reaction conditions to selectively obtain isolable aldehyde-terminated oligomers. Building on the optimized product selectivity and yield of the aldehyde end group, we explored the use of the degradation products by reacting them with diamine to build a cross-linked polymer network (Scheme 1). Specifically, we demonstrate the photo-oxidative degradation of unsaturated polymers into carbonyl-terminated oligomers under mild conditions (390 nm light, room temperature). As a proof of concept, postconsumer nitrile rubber (NBR) gloves underwent photo-oxidative cleavage to afford polymers bearing aldehyde end groups. These aldehyde-functionalized polymers were cross-linked with *p*-phenylenediamine (PH2NH) via dynamic imine bonds to form reconfigurable polymer networks. The resulting networks exhibited enhanced mechanical properties and hydrophobicity compared to the original waste rubber, achieving a closed-loop recycling system with performance parity to that of conventional polymeric materials. In addition, the aldehyde functional group in the degradation product can be converted into alcohol or carboxylic acid, allowing for a broader application of the photooxidative degradation product.

**Scheme 1:**
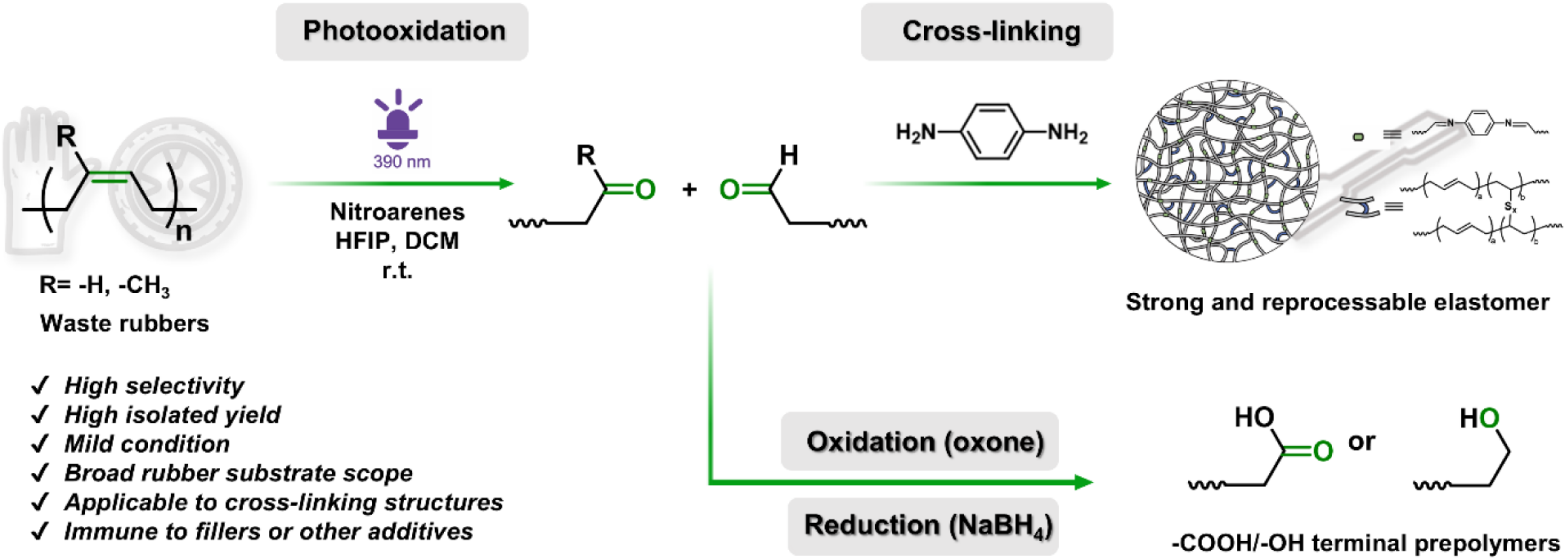
Schematic Strategy of a Photoexcited Nitroarene for Deconstruction and Upcycling of Unsaturated Rubbers

## Results and Discussions

### Model Reaction

Leonori and coworkers reported an efficient approach to achieving oxidative cleavage of alkenes using nitroarenes and purple-light irradiation at low temperature (−30 °C).^15^ Low temperature is a necessary reaction condition to achieve good conversion in the reported procedure. However, keeping a long-time low-temperature reaction is an energy-consuming process. In consideration of our target goal of highly selective acquisition of functionalized prepolymers instead of complete degradation, we started to perform model oxidation at room temperature. 2,2,2-Trifluoroethyl oleate (TFO) was selected as a model reactant due to TFO containing a methylene group close to −CF_3_, which is regarded as an internal standard to calculate olefin conversion and yield of aldehyde. As shown in Figure S1, nitrobenzene (NB), 4-nitrobenzonitrile (NBCN), 1,3-dinitro-5-(trifluoromethyl)­benzene (F2NB), and 1-nitro-3,5-bis­(trifluoromethyl)­benzene (2FNB) were used to test the oxidative cleavage of TFO into two aldehyde derivatives. Among the different nitroarenes, 2FNB displayed a high olefin conversion of 46.7% and kept an aldehyde yield of 32.4% at room temperature after optimization, as shown in Table S1. At the same time, 1,1,1,3,3,3-hexafluoropropan-2-ol (HFIP) was added into the oxidative system to slightly improve the aldehyde yield by reducing combination of hydrogen bonding to heteroatoms.^20^ As shown in Table S2, dichloromethane in photoexcited oxidation achieved a high conversion of 38.8% compared to other solvents. Then, we evaluated the oxidation of TFO under 365, 390, and 470 nm LED irradiation, as shown in Table S3. The results suggested that the aldehyde yield from TFO oxidative cleavage significantly increased from 7.7% (under 470 nm light) and 15.9% (under 365 nm light) to 27.0% (under 390 nm light). By the above optimization of reaction conditions, the successful oxidation of TFO using 390 nm light validates its potential use for the photomediated oxidative cleavage of unsaturated polymers.

### Photo-oxidative Deconstruction of Unsaturated Polymers

Having identified the primary results of the TFO oxidation by nitroarenes under 390 nm household LED irradiation, we investigated the oxidation of unsaturated polymers. Polybutadiene (PBD), a specific structure of mainly 98% cis olefin, was first selected to probe the photoexcited oxidation, as shown in Figure 1. As shown in Table 1, different nitroarenes including F2NB, NBCN, and 2FNB were used as oxidative agents to perform the photo-oxidation of PBD. An obvious insoluble product precipitated from the system (Table 1, entries 1–3 and Figure S2) using F2NB and NBCN as oxidative agents. Interestingly, this phenomenon was not observed in the case of 2FNB, and a high conversion of 28.5% (calculated by ^1^H NMR using mesitylene as the internal standard) was obtained. Based on these positive results, the photoexcited oxidation was optimized to obtain a high conversion of 33.7% and an aldehyde yield of 16.7%, as shown in Table 2, when the concentration of PBD was 40 mM and the ratio of PBD/2FNB/HFIP was 1/3/4. Subsequently, we also investigated the 100-time scale-up of the gram-level PBD conversion at the ratio of PBD/2FNB/HFIP of 1/1/1. The scale-up results showed that high oxidation conversion and aldehyde yield were kept, as shown in Table S4, entry 8. After oxidation, a significant status change in which the PBD bulk gel was converted into 2AD viscous liquid was observed, as shown in Figure 1a. At the same time, the oxidation results of PBD were confirmed by the appearance of the proton peak at 9.77 ppm (−CHO) in Figure 1b. In addition, the FT-IR spectrum of 2AD in Figure S10 showed the appearance of a new peak at 1724 cm^–1^ after oxidation, which can be attributed to the C=O stretching in aldehyde, further confirming the successful synthesis of 2AD. The photo-oxidative degradation was further confirmed by comparing the GPC traces of PBD and 2AD (Figure S8), as evidenced by the disappearance of the polymer peak at 106 kDa and the emergence of low molecular weight peaks at 5.9 kDa.

**Table 1:** Results of Photoexcited Oxidation of PBD by Different Nitroarenes (NAs)^a^

Run	NAs	PBD/NAs/HFIP^b^	Conv.^c^ (%)	Yield^d^ (%)	Yield^e^ (%)
1^f^	F2NB	1/1.5/0	45.9	9.3	65
2^f^	F2NB	1/3/4	n.d.	n.d.	n.d.
3^f^	NBCN	1/1.5/0	12.9	6.7	75
4	2FNB	1/1.5/0	10.0	8.5	72
5	2FNB	1/1/1	21.5	14.5	84
6	2FNB	1/3/4	28.5	15.6	76
7^g^	2FNB	1/3/4	33.7	16.7	86

a Oxidation was performed under 390 nm LED irradiation using different nitroarenes as photoexcited oxidants and DCM as the solvent at room temperature for 12 h. The concentration of PBD is 80 mM.

b The equivalents of NAs and HFIP were calculated relative to the molar amount of olefinic bonds in the polymer.

c As calculated through ^1^H NMR spectra using mesitylene as the internal standard.

d Yield of aldehyde as calculated through ^1^H NMR spectra using mesitylene as the internal standard.

e Isolated yield of 2AD.

f Partially insoluble products precipitated.

g The concentration of PBD is 40 mM.

**Figure 1:**
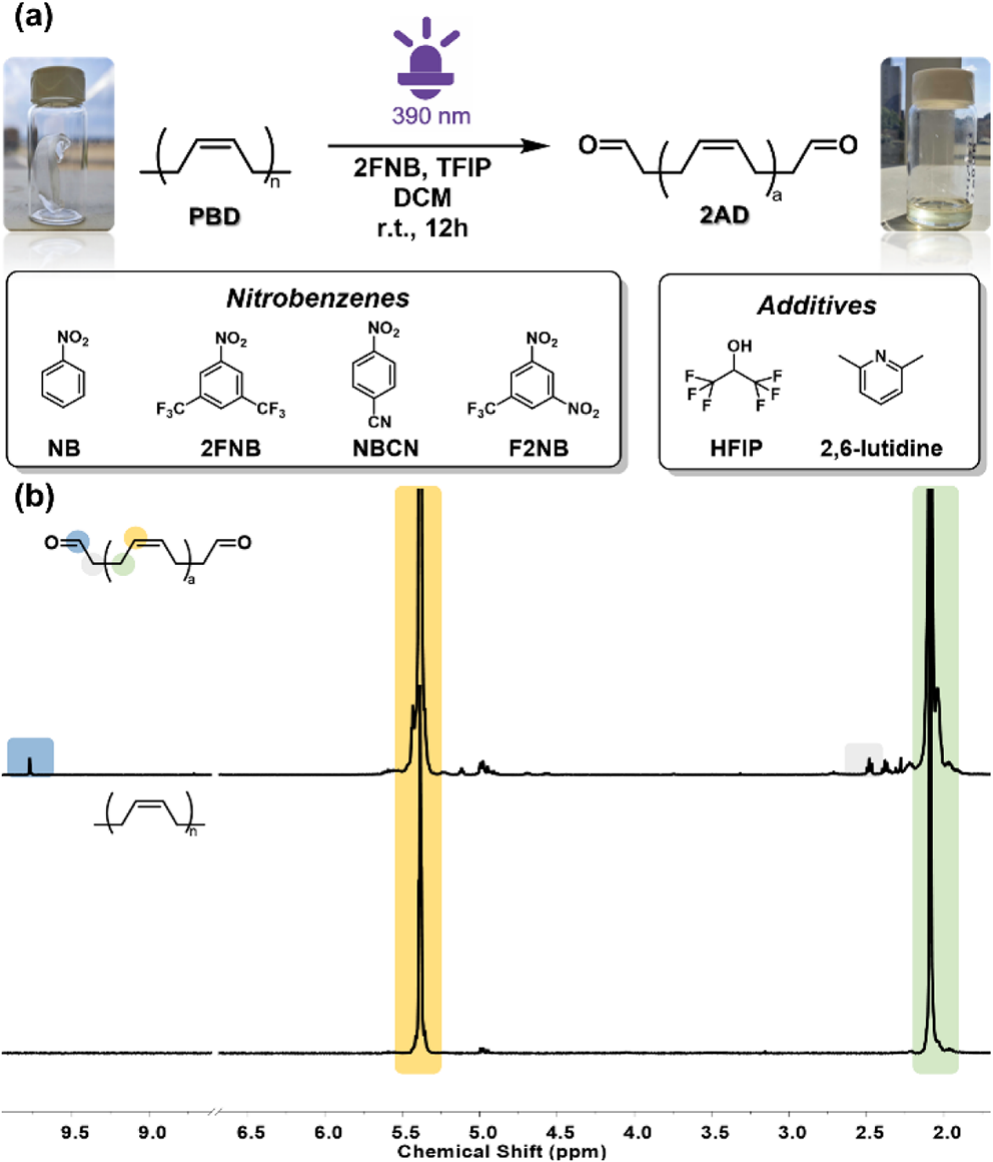
(a) The photoexcited oxidation of PBD under 390 nm LED irradiation. (b) ^1^H NMR of PBD and 2AD after purification in MeOH and vacuum drying.

**Table 2:**
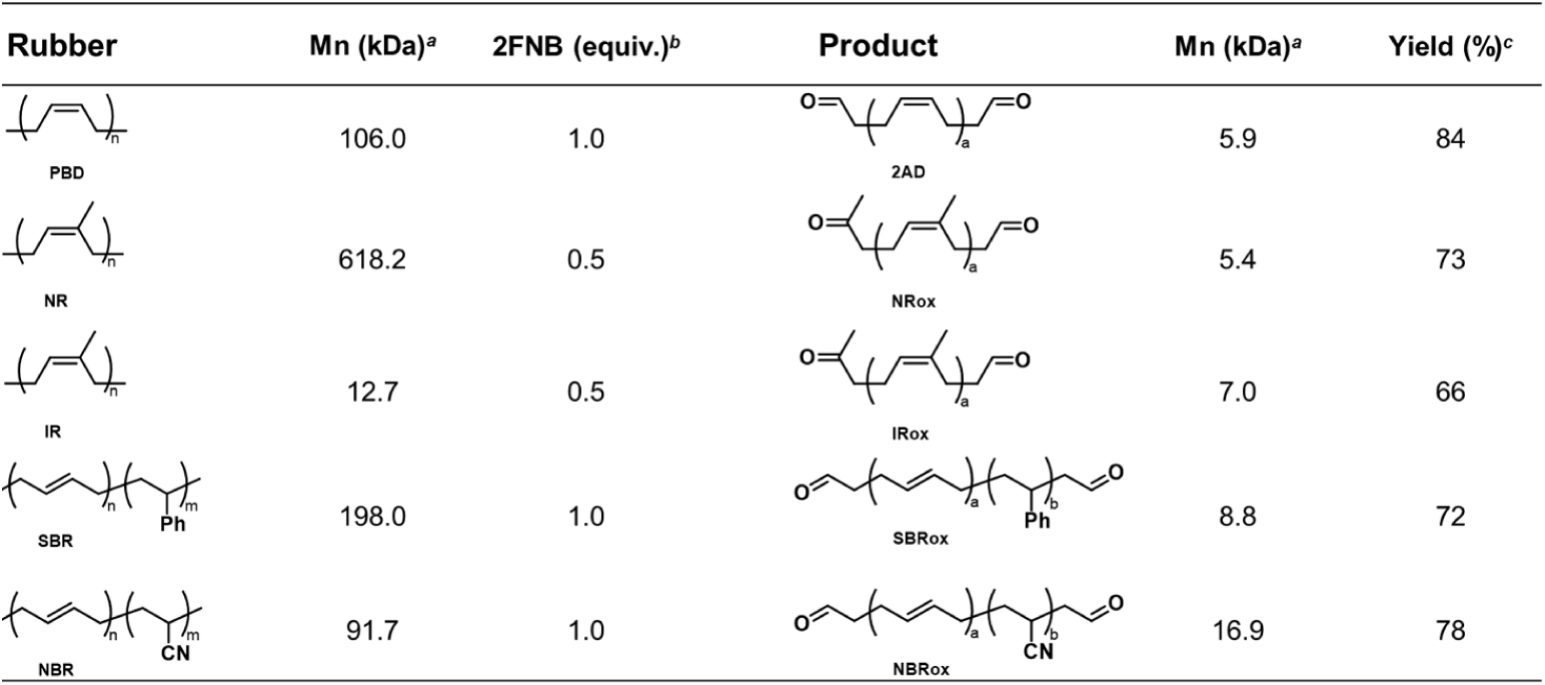
Results of Photoexcited Oxidation of Different Unsaturated Polymers^a^ ^b^ ^c^

a Molecular weight was determined by GPC at 40 °C in THF relative to polystyrene (PS) standards.

b Oxidation was performed by using 2FNB as the photoexcited oxidant and DCM as the solvent at room temperature for 12 h. The 2FNB equivalent was calculated relative to the molar amount of olefinic bonds in the polymer. The concentration of PBD is 80 mM.

c Isolated yield of the oxidation product.

Encouraged by the positive results of PBD oxidation, we extended this nitroarene oxidation approach to other commercially available unsaturated polymers to broaden its application scope. Natural rubber (NR), isoprene rubber (IR), styrene–butadiene rubber (SBR), and NBR were selected for photo-oxidation experiments, as these major commercial rubbers collectively constitute nearly all unsaturated rubber products.^21^ As shown in Table 2 and Figure S8, the molecular weights of various rubbers were reduced to low-molecular-weight prepolymers through photo-oxidative degradation. Notably, natural rubber, which accounts for nearly half of the annual global rubber production, demonstrated high degradation efficiency.^22^ In the presence of 0.5 equiv of 2FNB, its molecular weight dramatically decreased from 618.2 to 5.4 kDa, as shown in Figure S8. From the NMR results in Figure S4, the appearance of an aldehyde peak at 9.78 ppm and a methyl group adjacent to a carbonyl at 2.12 ppm indicates that the original olefin in NR was successfully oxidized to an aldehyde and ketone. The high degradation efficiency of NR can be attributed to the presence of a methyl group on the olefin, which facilitates the stable formation of N-doped ozonide five-membered ring intermediates through photoinduced cyclization between nitroarenes and olefin groups, as shown in Figure S9.^15^ In addition, SBR, widely used in tires, was efficiently degraded, with its molecular weight decreasing from 198.0 to 8.8 kDa. NBR, commonly used in gloves and sealing materials, was also degraded with a high isolated yield of 78%. These results demonstrate that the presence of pendant groups, such as phenyl in SBR and nitrile in NBR, does not significantly hinder nitroarene-mediated photo-oxidation. This confirms the method’s versatility as a universal platform for deconstructing major unsaturated rubbers under mild conditions.

Based on the positive results of NR degradation, a reaction mixture containing NBR, 2FNB, and HFIP was exposed to light, stirred in the dark for a predetermined time, and then re-exposed to light to investigate the kinetics of photoexcited oxidation (Figure 2). The yield of aldehyde groups and the molecular weight were monitored at each light–dark switching point by using NMR spectroscopy and GPC analyses of aliquots. As shown in Figure 2a and summarized in Table S5, the plot of aldehyde yield versus time demonstrates that photo-oxidation occurred only under light exposure in the presence of 2FNB. Furthermore, the GPC analysis of the NR degradation products (Figure 2b and Table S5) revealed that the molecular weight remained unchanged during dark periods (e.g., between 60 and 90 min and 120–180 min), confirming that chain scission requires light irradiation. However, upon light exposure, the degradation curve shifted toward lower molecular weights. After irradiation was completed with a 390 nm household LED, the molecular weight of NR decreased to below 2.4 kDa. To gain deeper insight into the degradation mechanism, the kinetic data were fitted to eq 1 (presented in Figure 2c), as proposed by the Ebert group.^23^ The fitting of our photomediated degradation system yielded an α value of 0.67 (Figure 2c), indicating that the degradation process closely approximates random scission.

**Figure 2:**
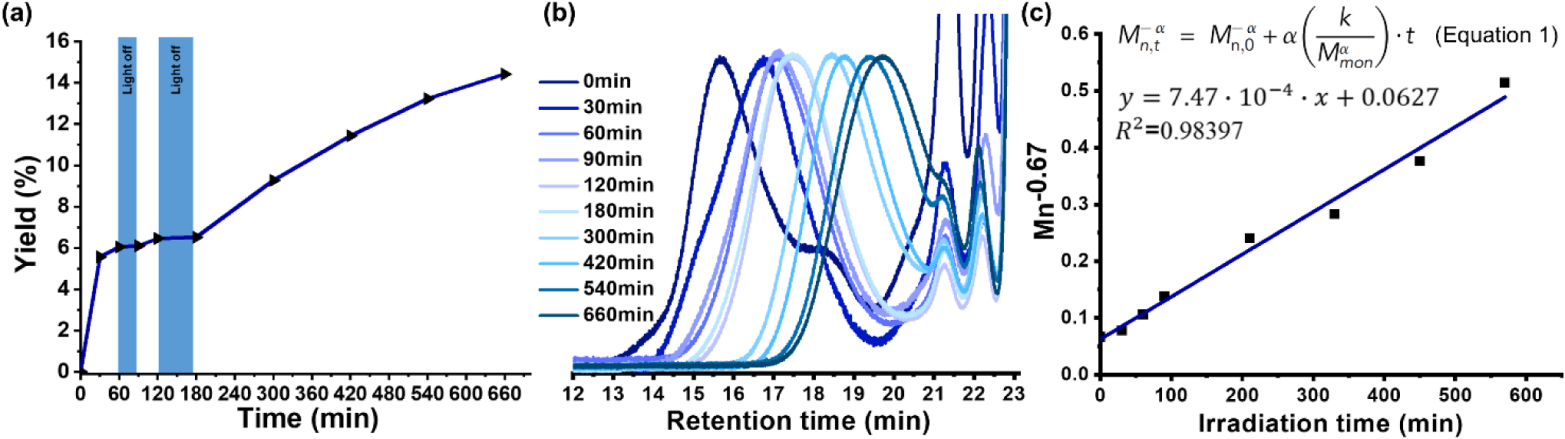
Kinetics results of NR photo-oxidation (1.6 g of NR, [olefin] = 0.15 M, 1 equiv of 2FNB, 1 equiv of HFIP, 390 nm LED irradiation at room temperature). (a) Plot of aldehyde yield against time. (b) GPC traces of NR oxidative degradation at different times. (c) Plot of Mn^0.67^ against irradiation time.

### Photo-oxidative Deconstruction and Upcycling of Postconsumer NBR Gloves

Vulcanization is the primary method for enhancing the mechanical strength and antiaging properties of commercial rubber. However, highly cross-linked vulcanized rubber poses challenges for material degradation and recyclability.^3^ To address this issue, we investigated the photochemical degradation of NBR gloves, a common unsaturated rubber product in our laboratory, using nitroarene as an oxidant. Waste nitrile gloves were cut into small pieces and added to a DCM solution containing 2FNB as the oxidant and HFIP as an additive. After 48 h of irradiation at 390 nm, the waste NBR gloves dispersed into the DCM solution, as shown in Figure S3. The crude product was subsequently processed and purified, yielding a regenerated viscous NBR gel (NBR GLox) in a high yield of 63% (Figure 3a). FT-IR spectra of NBR GLox displayed a significant aldehyde signal at 1724 cm^–1^, while NMR analysis revealed an aldehyde peak at 9.77 ppm (Figure 3b and 3c). Furthermore, GPC analysis (Figure S14) exhibited a single peak at 18.2 min (*M*_n_ = 17.5 kDa). These results confirm the occurrence of photo-oxidative degradation of commercial NBR gloves, leading to the selective regeneration of NBR gel. Therefore, photodegradation using nitroarene as an oxidant presents an efficient strategy for degrading vulcanized rubber without being hindered by cross-linking.

**Figure 3:**
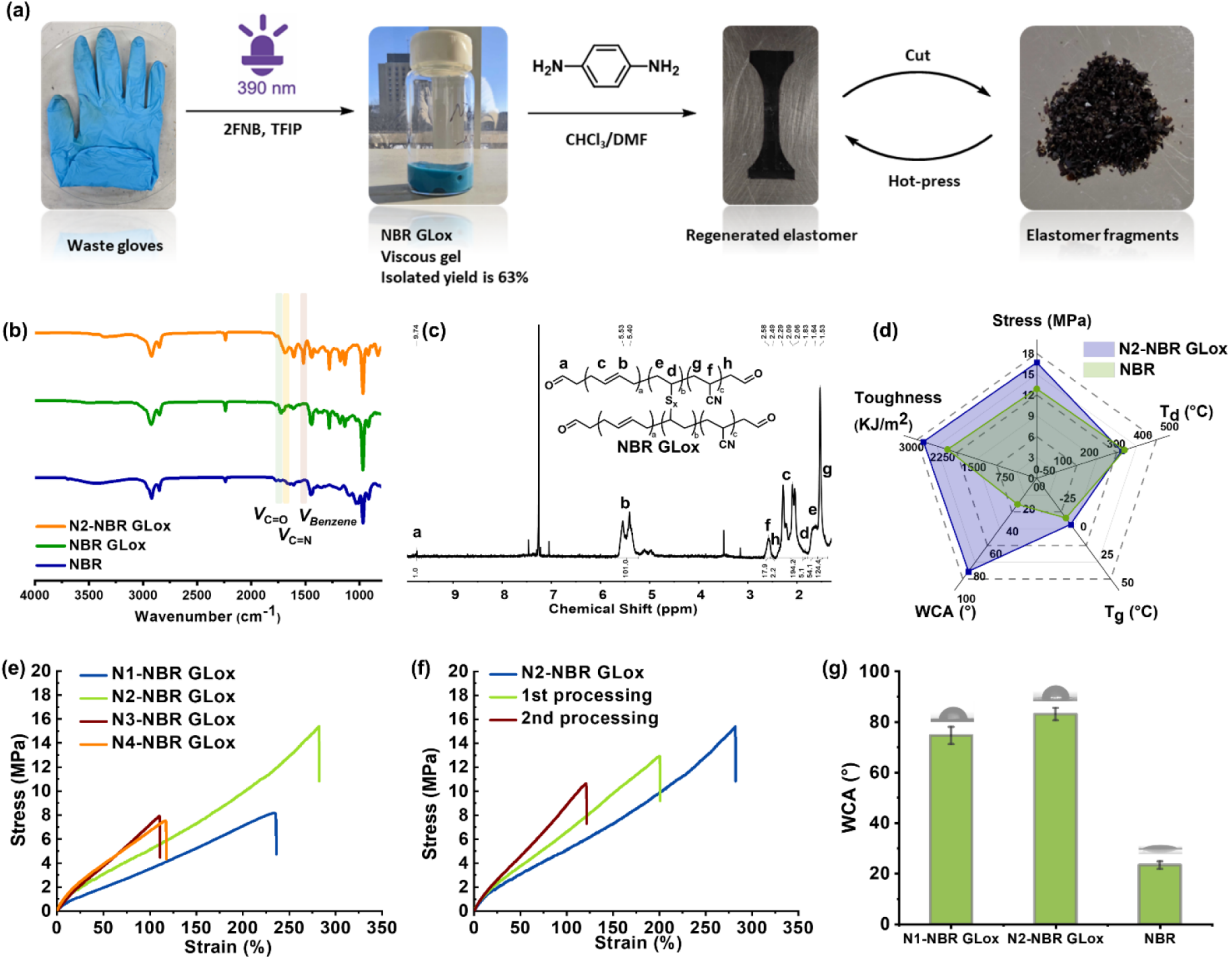
Photochemical recycling of waste NBR gloves. (a) Schematic of nitroarene-mediated photodegradation of NBR gloves and subsequent fabrication of reprocessable elastomers via imine dynamic networks. (b) FT-IR spectra of pristine NBR, oxidized degradation products (NBR GLox), and imine networks constructed from NBR GLox (N2-NBR GLox). (c) ^1^H NMR spectrum of NBR GLox after purification by MeOH. (d) Comparative properties of commercial NBR vs dynamic imine networks derived from NBR GLox: glass transition temperature (*T*_g_), thermal degradation onset (*T*_d_), tensile strength, toughness, and water contact angle (WCA). (e) Stress–strain curves of imine networks with varying NBR GLox/PH2NH ratios. (f) Reprocessability assessment: Stress–strain curves after two recycling cycles. (g) WCA comparison between regenerated networks and commercial NBR.

Leveraging the well-defined aldehyde intermediates, we hypothesized that the regenerated gel from waste rubber performed simple cross-linking to achieve repossessing and recycling of the elastomer. As shown in Figures 3a and S15, PH2NH was selected as the cross-linking agent to construct dynamic networks by simple blending without a catalyst and heat dehydration in a vacuum. From FT-IR spectra (Figure 3b), the original aldehyde signal at 1724 cm^–1^ disappeared and the characteristic peak of the imine (C=N) bond at 1684 cm^–1^ appeared at the same time, suggesting the accomplishment of cross-linking. Optimizing the ratio of NBR Glox and PH2NH as shown in Table S6 was performed for various properties. Notably, elastomers synthesized from waste glove degradation products exhibit a 29% higher tensile strength (12.9 → 16.7 MPa) and 27% greater toughness (2.15 → 2.73 MJ/m^3^) versus virgin NBR gloves (Figures 3d and S16–S18). This mechanical enhancement is attributed to aromatic hard segments and dynamic imine bonds within the polymer network.^24^ Furthermore, surface hydrophobicity significantly increased, with water contact angles rising from 23 ± 2° to 83 ± 3° (Figure 3g), resulting from the introduction of hydrophobic aromatic cross-linkers. As shown in Figure S19, the network demonstrated good stability against hydrolysis. After 21 days, the mass loss in all tested media was negligible, remaining below 5%. Furthermore, visual inspection (Figure S19, inset) confirmed that the samples retained their structural integrity, with no significant shape change after the 21-day hydrolysis period. This enhanced stability is likely attributed to the surface hydrophobicity significantly increased within the system, which helps shield the dynamic imine bonds from the aqueous environment. Enhancing the hydrophobic property and hydrolysis stability of rubber may improve chemical resistance and mitigate swelling phenomena, thereby prolonging the service life of the rubber in potential applications such as industrial cables and sealing components.^25^

Subsequently, we cut the N2-NBR GLox into small pieces and used hot pressing (30 MPa, 60 min) to test the processable properties. The results showed that N2-NBR GLox with chemical cross-links can be effectively processed and yield a defect-free film under hot processing conditions. Among them, the tensile strengths of the reprocessed N2-NBR GLox after two cycles of hot pressing had corresponding tensile strength recovery ratios of 83.8% and 68.8%, respectively. Accordingly, the elastic modulus recycling efficiency of the reprocessed N2-NBR GLox after two cycles of hot pressing was increased by 73.3% and 74.5%, respectively. These are primarily attributed to the formation of permanent cross-links through side reactions, such as self-cross-linking and hyper-cross-linked polyamine under repeated thermal reprocessing conditions.^26,27^ The thermal properties of N2-NBR GLox and original NBR were determined by TGA and DSC, as shown in Figures S20 and S21. The glass transition temperatures of the regenerated network and commercial rubber are close: −8.8 °C vs −14.5 °C. The degradation temperatures *T*_d_ (defined by the temperature at which 5% weight loss occurs) of dynamic networks and NBR gloves were 318 and 330 °C, respectively. Therefore, the above results showed that the improvement of mechanical and surface properties was achieved by imine dynamic chemistry without the obvious loss of thermal properties (Figure 3d).

### Functional Transformation of the Aldehyde-Terminated Oligomers from Photo-oxidation

In addition to direct use, the aldehyde functional group can also be conveniently converted into a carboxylic acid or hydroxy group (Figure 4) to further improve the value of photo-oxidation-degraded products. First, the aldehyde group of 2AD was transformed into a carboxylic acid terminal prepolymer in the presence of oxone, a commercial and safe oxidative.^28,29^ As shown in Table S7 and Figure S22, the component of the solvent plays a significant role in selective oxidation from aldehyde to −COOH. The incorporation of water resulted in the epoxidation of olefins instead of the oxidation of the aldehyde. Notably, when using the cosolvent of DMF and THF, 2AD was successfully oxidized into a carboxyl terminal unsaturated prepolymer (2AD-COOH) with a high conversion of 91%. Having identified the optimal reaction conditions, we demonstrated the scale-up of further oxidation conversion (Table S7, entry 5). A high conversion of 93% was kept with a prolonged reaction time from 3 to 12 h. The oxidation result was identified by NMR. The original aldehyde peak of 2AD at 9.77 ppm disappeared, while a new signal attributed to methylene adjacent to the carboxylic acid of 2AD-COOH at 2.39 ppm was observed in Figure S23.

**Figure 4:**
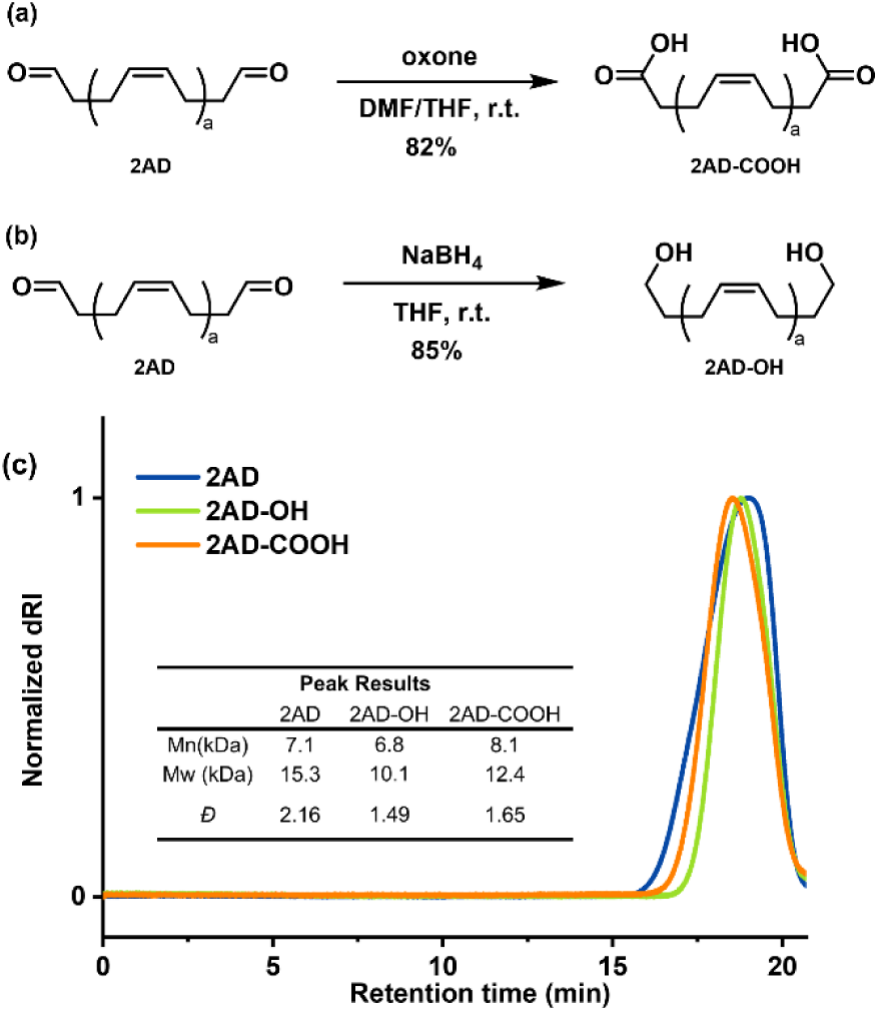
(a) Further oxidation of the 2AD prepolymer to obtain 2AD-COOH. (b) Reduction of the 2AD prepolymer to obtain 2AD–OH. (c) GPC traces of 2AD, 2AD–OH, and 2AD-COOH.

Then, we performed the transformation of 2AD into the hydroxy-terminal unsaturated prepolymer (2AD–OH) (Figure 4b). As shown in Table S8, the aldehyde group was smoothly reduced to 2AD–OH using an excess of NaBH_4_ with a high conversion. The structural transformation was identified by NMR from the complete disappearance of aldehyde chemical shifts and the appearance of a new signal at 3.64 ppm assigned to the methylene close to the hydroxyl group. In addition, the results that the aldehyde band at 1724 cm^–1^ obviously vanished after reduction also suggested that 2AD–OH was successfully synthesized, as shown in Figure S10. We also used GPC to determine the change of molecular weight in the process of terminal functional group transformation (Figure 4c). No obvious decrease was observed in either further oxidation or reduction. This suggested that no chain scission or intermolecular condensation occurred, which also implied good selectivity of the product. The incorporation of carboxylic acid and hydroxyl groups into unsaturated polymers offers promising applications in the development of novel vitrimers and polymeric additives, including curing agents, chain extenders, and toughening agents.^30^

## Conclusions

This study establishes a photochemical oxidation strategy for the efficient degradation of unsaturated polymers. The method operates under mild conditions with high generality and selectivity, overcoming key recycling barriers: additive interference (fillers/flame retardants) and cross-linked network stability. Demonstrated through NBR glove upcycling, this process yields processable aldehyde-functionalized gels. Subsequent dynamic imine chemistry with diamines transforms these intermediates into robust hydrophobic elastomers with enhanced mechanical properties and surface characteristics. This structural reorganization valorizes waste into high-performance materials, creating economically viable pathways for sustainable circularity. Furthermore, the versatile aldehyde intermediates can be selectively oxidized to carboxylic acids or reduced to hydroxyl groups, enabling utility as multifunctional polymeric additives (curing agents, chain extenders, tougheners) beyond elastomer synthesis.

## Supplementary Material



## Notes

All data necessary to support the conclusions of this paper, including the experimental section, supplementary figures and tables, NMR, FT-IR, DSC, TGA, and GPC results, additional references (PDF)

## References

[1] Malaysian Rubber Council. Rubber Industry Overview; 2024, https://www.myrubbercouncil.com/industry/world_production.php?utm_source.

[2] Towell S. E., Ratushnyy M., Cooke L. S., Lewis G. M., Zhukhovitskiy A. V. (2025). Deconstruction
of rubber via C–H amination and aza-Cope rearrangement. Nature.

[3] Wu P., Hu Q., Ogunfowora L. A., Li Z., Marquardt A. V., Savoie B. M., Dou L. (2025). Toward Sustainable
Polydienes. J. Am. Chem. Soc..

[4] Wu P., Hu Q., Marquardt A. V., Ogunfowora L. A., Kim J. H., Tang Y., Lin C., Savoie B. M., Dou L. (2025). Photoinduced bulk polymerization
strategy in melt state for recyclable polydiene derivatives. Nat. Chem..

[5] Hu Q., Luo X., Ogunfowora L. A., Athaley A., DesVeaux J. S., Klein B. C., Xu S., Wu P., Wei Z., Lin C. (2025). Scalable, biologically sourced depolymerizable polydienes
with intrinsically weakened carbon–carbon bonds. Nat. Chem. Eng..

[6] Britt, P. F. ; Coates, G. W. ; Winey, K. I. ; Byers, J. ; Chen, E. ; Coughlin, B. ; Ellison, C. ; Garcia, J. ; Goldman, A. ; Guzman, J. Report of the Basic Energy Sciences Roundtable on Chemical Upcycling of Polymers; U.S. Department of Energy, 2019, 10.2172/1616517.

[7] Bekmirzaev J., Simon M., D’Aniello S., Mazzeo M., Cohen-Janes S. J., Mathers R. T., Gauvin R. M., Thomas C. M. (2024). A New Life For Nitrile-Butadiene
Rubber: Co-Harnessing Metathesis And Condensation For Reincorporation
Into Bio-Based Materials. Angew. Chem., Int.
Ed..

[8] Park B., Cho K., Choi K., Hong S. H. (2025). Catalytic and selective chemical
recycling of post-consumer rubbers into cycloalkenes. Chem.

[9] Sathe D., Zhou J., Chen H., Su H.-W., Xie W., Hsu T.-G., Schrage B. R., Smith T., Ziegler C. J., Wang J. (2021). Olefin metathesis-based
chemically recyclable polymers enabled by
fused-ring monomers. Nat. Chem..

[10] Chen H., Guan X., Zhang P., Sathe D., Wang J. (2024). Deconstruction
of unsaturated polymers through photo-mediated oxidation under O_2_. Cell Rep. Phys. Sci..

[11] Kim D., Yu C., Kwon M. S. (2025). Degradable
Adhesives as Sustainable Alternatives to
Acrylics via Ring-Opening Radical Polymerization of Vinylcyclopropanes. Angew. Chem., Int. Ed..

[12] Korpusik A. B., Adili A., Bhatt K., Anatot J. E., Seidel D., Sumerlin B. S. (2023). Degradation of Polyacrylates by One-Pot Sequential
Dehydrodecarboxylation and Ozonolysis. J. Am.
Chem. Soc..

[13] Berto P., Mehats J., Wirotius A.-L., Grelier S., Peruch F. (2022). Reprocessable
Covalent Elastomeric Networks from Functionalized 1,4-cis-Polyisoprene
and -Polybutadiene. Macromolecules.

[14] Berto P., Pointet A., Le Coz C., Grelier S., Peruch F. (2018). Recyclable
Telechelic Cross-Linked Polybutadiene Based on Reversible Diels–Alder
Chemistry. Macromolecules.

[15] Ruffoni A., Hampton C., Simonetti M., Leonori D. (2022). Photoexcited nitroarenes
for the oxidative cleavage of alkenes. Nature.

[16] Wise D. E., Gogarnoiu E. S., Duke A. D., Paolillo J. M., Vacala T. L., Hussain W. A., Parasram M. (2022). Photoinduced Oxygen Transfer Using
Nitroarenes for the Anaerobic Cleavage of Alkenes. J. Am. Chem. Soc..

[17] Hampton C., Simonetti M., Leonori D. (2023). Olefin Dihydroxylation Using Nitroarenes
as Photoresponsive Oxidants. Angew. Chem., Int.
Ed..

[18] Miyake G., Liu X., Hu Z., Portela B. S., Rettner E. M., Pineda A., Miscall J., Rorrer N. A., Krummel A. T., Paton R. S. (2024). Photooxidation
of Polyolefins to Produce Materials with In-chain Ketones and Improved
Materials Properties. Angew. Chem., Int. Ed..

[19] Le T. H., Bhatt K., Dworakowska S., Stewart K. A., Boeck P. T., Veige A. S., Seidel D., Sumerlin B. S. (2025). Nitroarene Photoactivation
Promotes Oxidative Deconstruction of Olefinic Polymers. ACS Macro Lett..

[20] Bietti M. (2018). Activation
and deactivation strategies promoted by medium effects for selective
aliphatic C– H bond functionalization. Angew. Chem., Int. Ed..

[21] Vijayaram T. R. (2009). A technical
review on rubber. Int. J. Des. Manuf. Technol..

[22] Mathew A.
M. (2020). Inclinations
and challenges of global rubber production. PBME.

[23] Basedow A.
M., Ebert K. H., Ederer H. J. (1978). Kinetic Studies on the Acid Hydrolysis
of Dextran. Macromolecules.

[24] Guo M., Huang Y., Chen Z., Zhang Y., Zhang Y., Zhu M., Zhang J., Feng S. (2021). Preparation and Properties of Benzylsulfonyl-Containing
Silicone Copolymers via Ring-opening Copolymerization of Macroheterocyclosiloxane
and Cyclosiloxane. Chem.—Eur. J..

[25] Atthi N., Nimittrakoolchai O., Jeamsaksiri W., Supothina S. (2008). Chemical resistant
improvement of natural rubber and nitrile gloves by coating with hydrophobic
film. Adv. Mater. Res..

[26] Wu J.-N., Chen L., Fu T., Zhao H.-B., Guo D.-M., Wang X.-L., Wang Y.-Z. (2018). New application
for aromatic Schiff
base: High efficient flame-retardant and anti-dripping action for
polyesters. Chem. Eng. J..

[27] Jia Z., Wang H., Yu P., He H., Huang Q., Hong W., Liu C., Shi Y., Wang J., Xin Y. (2024). Soft-Rigid Construction
of Mechanically Robust, Thermally
Stable, and Self-Healing Polyimine Networks with Strongly Recyclable
Adhesion. Small.

[28] Yu B., Liu A.-H., He L.-N., Li B., Diao Z.-F., Li Y.-N. (2012). Catalyst-free approach for solvent-dependent selective oxidation
of organic sulfides with oxone. Green Chem..

[29] Hussain H., Green I. R., Ahmed I. (2013). Journey Describing
Applications of
Oxone in Synthetic Chemistry. Chem. Rev..

[30] Kébir N., Campistron I., Laguerre A., Pilard J.-F., Bunel C., Couvercelle J.-P., Gondard C. (2005). Use of hydroxytelechelic cis-1,4-polyisoprene
(HTPI) in the synthesis of polyurethanes (PUs). Part 1. Influence
of molecular weight and chemical modification of HTPI on the mechanical
and thermal properties of PUs. Polymer.

