# Supplementary material for: Nitroarene-Mediated Photo-oxidative Deconstruction and Upcycling of Unsaturated Rubber Waste
Source: Macromolecules. 2026 Jan 22;59(3):1119–25. doi: 10.1021/acs.macromol.5c02306 (PMC12895529; doi:10.1021/acs.macromol.5c02306)
Supplement: Supplementary file 1 [file ma5c02306_si_001.pdf]

Supporting Information

Nitroarene-Mediated Photo-Oxidative Deconstruction and Upcycling of Unsaturated Rubber Waste

Mengdong Guo, Zefeng Wang, Roberto Obregon, Obed Fernando, Junpeng Wang*

School of Polymer Science and Polymer Engineering, The University of Akron, 170 University Ave, Akron, Ohio 44325, United States

Table of Contents

1. Materials and Methods 3
2. General Procedures 4
Figure S1. The model reaction of TFO oxidation under 390 nm LED irradiation. 5
Figure S2. Photographs of photoexcited oxidation of PBD by F2NB and NBCN [a] 7
Figure S3. Photographs of photoexcited oxidation of waste gloves by F2NB 8
Table S1. Results of photoexcited oxidation of TFO by different nitroarenes (NAs) [a] 11
Table S2. Results of photoexcited oxidation of TFO by using different solvent [a] 11
Table S3. Results of photoexcited oxidation of TFO under different wavelength LED irradiation [a] 12
Table S4. Optimized results of photoexcited oxidation of PBD [a] 13
Figure S4. ^{1}H NMR spectrum of NR and NRox 13
Figure S5. ^{1}H NMR spectrum of IR and IRox 14
Figure S6. ^{1}H NMR spectrum of SBR and SBRox 15
Figure S7. ^{1}H NMR spectrum of NBR and NBRox 15
FigureS8. GPC traces of different unsaturated polymers and photo-oxidation products 16
Figure S9. Proposed mechanism of NR photochemical degradation 16
Figure S10. FT-IR spectra of PBD and 2AD and 2AD-OH and 2AD-COOH 17
Figure S11. FT-IR spectra of NR and NRox 17
Figure S12. FT-IR spectra of SBR and SBRox 18
Figure S13. FT-IR spectra of NBR and NBRox 18
Table S5. Kinetics results of NR photo-oxidation 19
Figure S14. GPC traces of NBR GLox 19
Figure S15. Scheme of construction network form NBR Glox with PH2NH 20
Table S6. Optimized results of different ratio of NBR GLox and PH2NH to construct networks 20
Figure S16. Stress-strain curves of N2-NBR GLox 21
Figure S17. Stress-strain curves of commercial NBR 21
Figure S18. Mechanical properties of stress and toughness 22
Figure S19. Degradation profiles of N2-NBR GLox in different aqueous media 22
Figure S20. TGA curves of NBR and N2-NBRGLox in nitrogen 23
Figure S21. DSC traces of NBR and N2-NBRGLox in nitrogen 23
Table S7. Optimized results of different ratio of further oxidation from 2AD into 2AD-COOH [a] 24
Figure S22. ^{1}H NMR spectrum of 2AD-COOH using different solvents 24
Figure S23. ^{1}H NMR spectrum of 2AD-COOH 24
Table S8. Optimized results of reduction of 2AD prepolymer to obtain 2AD-OH [a] 25
Figure S24. ^{1}H NMR spectrum of 2AD-OH [a] 25
References 26

1. Materials and Methods

1.1 Materials

Nitrobenzene, 4-nitrobenzonitrile, polybutadiene (PBD, 98% cis, M_w = 200000~300000), polyisoprene (IR, M_w = 38000), 1,4-phenylenediamine, tris(2-aminoethyl)amine, were purchased from Sigma-Aldrich. Oleic acids were purchased from Alfa Aesar. 2,6-Lutidine, 1,3-dinitro-5-(trifluoromethyl)benzene and N-phenylmaleimide were purchased from TCI. 1-Nitro-3,5-bis(trifluoromethyl)benzene were purchased from Ambeed. 1,1,1,3,3,3-Hexafluoropropan-2-ol were purchased from oakwood. All solvents were purchased from Fisher. Styrene-butadiene rubber (SLF® 16S42, vinyl content 42%, styrene content 16%) (SBR) were provided from Goodyear Chemicals. Commercial nitrile gloves was purchased from USA GLOVES. Natural rubber (NR) was purchased from Aaron Chemicals. Poly(acrylonitrile-co-butadiene) (acrylonitrile 37-39wt%) (NBR) were purchased from Aladdin Chemicals. 2,2,2-Trifluoroethyl oleate (TFO) was prepared following the previous reported procedure [1].

1.2 Characterization

Fourier transform infrared spectra (FT-IR) were recorded on a Perkin Elmer infrared spectrophotometer equipped with attenuated total reflection accessory from 4000 cm^{-1} to 400 cm^{-1}. Nuclear magnetic resonance (^1H NMR and ^{13}C NMR) spectra using deuterated chloroform ($CDCl_3$) as a solvent were performed with a Ailgent 500 spectrometers under room temperature. Gel permeation chromatograph (GPC) measurements for polymers and degraded products were carried out in THF (1.0 mL min^{-1}) at 313 K using polystyrene (PS) as the standard. The GPC was equipped with a Tosoh EcoSEC HLC-8320GPC with two 17393 TSK gel columns (7.8 mm ID × 30 cm, 13 μm) and one 17367-TSK gel guard column (7.5 mm ID × 7.5 cm, 13 μm). Tensile testing was performed using an Instron 5969 tensile tester with a 1 kN load cell controlled by an Arduino UNO at strain rate of 5%/s. Thermogravimetric analysis (TGA) was measured by a TA

Discovery TGA 550 in nitrogen (20 mL min^{-1}) at a heating rate of 10 °C min^{-1} from room temperature to 800 °C. Differential scanning calorimetry (DSC) was performed with a TA Discovery DSC 250 instrument and the samples were loaded in aluminum pans, heated from -90 to 100 °C. The heating and cooling temperature ramping rates were 10 °C min^{-1}. Water contact angles were measured using a Ramé-Hart Model 500-F1 Advanced Goniometer equipped with DROPimage Advanced software (Ramé-Hart Instrument Co., USA). For each substrate, ≥3 measurements were performed at different surface locations. Reported values represent mean ± standard error of the mean (s.e.m.).

2. General Procedures

2. 1 A representative procedure of photo-excited nitroarenes for TFO oxidation

The photoexcited oxidation of model reagent TFO was performed by nitroarenes under 390 nm (Figure S1). To a 20 mL vial, TFO (0.44 mmol, 1 equiv), 2FNB (0.34 g, 1.32 mmol, 3 equiv) and HFIP (0.30 g, 1.77mmol, 4 equiv) were dissolved in 1.34 mL DCM. After purging with nitrogen, the vial was stirring and irradiated under 390 nm household LEDs for 12h. The fan was turned on to keep the vial at room temperature. Upon completion of the irradiation, the reaction mixture was treated with 12 mL of a freshly prepared cocktail comprising N-phenylmaleimide (76 mg, 0.44 mmol, 1 equiv) and 39% aqueous formaldehyde (188 μL, 0.44 mmol, 6 equiv) in a CH_3CN/H_2O (3:1, v/v) mixture. The obtained mixture was stirring for 6 h at room temperature. After that, the product was washed by 20 mL brine and 40 mL DCM. The aqueous phase was extracted with DCM. The organic phase was dried over anhydrous Na_2SO_4 and evaporated. The dried mixture was then directly characterized by ^1H NMR to determine the conversion of alkene and yields of aldehyde groups. Protons in the trifluoroethyl group at 4.45 ppm were used as internal standard for calculating conversions and yield of aldehyde. Conversion was calclulated by Conv.% = ($I_{4.45ppm}$ - $I_{5.35ppm}$) / $I_{4.45ppm}$ * 100%. Yield of aldehyde was calculated by Yield% = $I_{9.77ppm}$ / $I_{4.45ppm}$ * 100%.

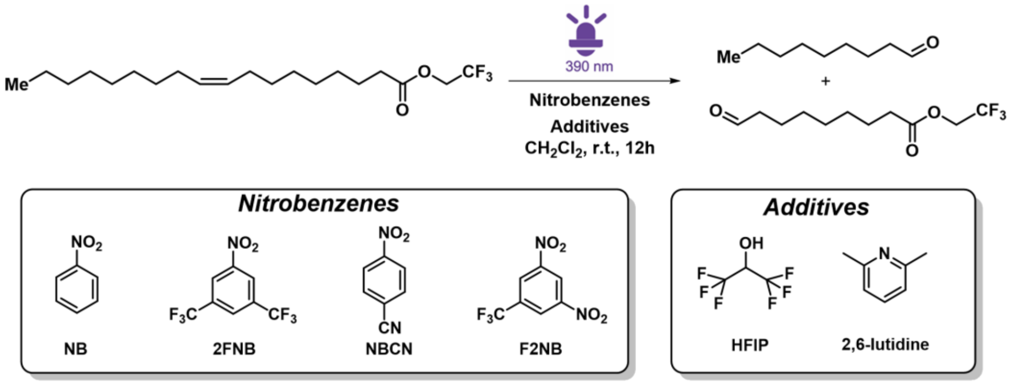

Figure S1. The model reaction of TFO oxidation under 390 nm LED irradiation.

2. 2 A representative procedure of photo-excited oxidation for unsaturated polymers

The photoexcited oxidation of polybutadiene was performed by 2FNB under 390 nm. A 20 mL vial was charged with PBD (43 mg, 0.80 mmol, calculated relative to the molar amount of olefinic bonds in the polymer, 1 equiv), 2FNB (210 mg, 0.80 mmol, 1 equiv), HFIP (130 mg, 0.80 mmol, 1 equiv), and DCM (4.3 mL). The solution was purged with nitrogen for 15 minutes and then irradiated with 390 nm household LEDs while being vigorously stirred for 12 hours. Throughout the process, a fan was used to maintain the reaction temperature at ambient conditions. Upon completion of the irradiation, the reaction mixture was treated with 4 mL of a freshly prepared cocktail comprising N-phenylmaleimide (138 mg, 0.80 mmol, 1 equiv) and 39% aqueous formaldehyde (342 µL, 4.80 mmol, 6 equiv) in a CH_3CN/H_2O (3:1, v/v) mixture. After stirring at room temperature for 6 hours, the mixture was worked up by washing with brine (20 mL) and DCM (40 mL). The aqueous phase was extracted with DCM, and the combined organic phases were dried over anhydrous Na_2SO_4 and evaporated under reduced pressure. The product was then submitted to ^1H NMR analysis after the addition of mesitylene (96 mg, 0.80 mmol, 1 equiv) as an internal standard to calculate the conversion and yield of aldehyde. The conversion of alkenes and

yield of aldehyde was calculated using the phenyl proton resonance of mesitylene at 6.80 ppm as the reference. Conversion was calculated by Conv.% = ($I_{6.80ppm}$ − 1.5$I_{5.43ppm}$) / $I_{6.80ppm}$ * 100%; Yield of aldehyde was calculated by Yield% = 1.5$I_{9.77ppm}$ / $I_{6.80ppm}$ * 100%. The oxidative mixture was precipitated in cold methanol to afford a pale-yellow liquid. The obtained liquid was placed in vacuum overnight to calculate isolated yield of 86%. ^{1}H NMR (500 MHz, Chloroform-*d*) δ 9.77 (t, 1H, −CH_2−C*H*O), 5.39 (m, 2aH, −CH_2−C*H*=C*H*−CH_2−), 2.48 (t, *J* = 7.3 Hz, 2H, −CH_2−CHO), 2.37 (t, *J* = 7.2 Hz, 2H, −CH_2−CH_2−CHO), 2.08 (m, 4aH, −CH_2−CH=CH−). FT-IR (ν, cm^{-1}): 1728 (-C*H*O); M_n (GPC) = 5.9 kDa, *Đ*=1.8.

The oxidative degradation of NR (NRox): Yield = 73%. ^{1}H NMR (500 MHz, Chloroform-*d*) δ 9.77 (t, 1H, −CH_2−C*H*O), 5.13 (m, aH, −CH_2−C(CH_3) =C*H*−CH_2−), 2.48 (t, *J* = 7.3 Hz, 2H, −CH_2−CHO), 2.43 (t, 2H, −CH_2−CH_2−C=O(CH_3)), 2.35 (t, *J* = 7.2 Hz, 2H, −CH_2−CH_2−CHO), 2.26 (t, 2H, −CH_2−CH_2−C=O(CH_3)), 2.12 (m, 3H, −CH_2−CH_2−C=O(CH_3)), 2.04 (m, 4aH, −CH_2−C(CH_3)=CH− CH_2−), 1.68 (m, 3aH, −CH_2−C(CH_3)=CH−CH_2−). FT-IR (ν, cm^{-1}): 1724 (-C=O); M_n (GPC) = 5.4 kDa, *Đ* = 1.6.

The oxidative degradation of IR (IRox): Yield = 66%. ^{1}H NMR (500 MHz, Chloroform-*d*) δ 9.77 (t, 1H, −CH_2−C*H*O), 5.13 (m, aH, −CH_2−C(CH_3) =C*H*−CH_2−), 2.48 (t, *J* = 7.3 Hz, 2H, −CH_2−CHO), 2.43 (t, 2H, −CH_2−CH_2−C=O(CH_3)), 2.35 (t, *J* = 7.2 Hz, 2H, −CH_2−CH_2−CHO), 2.25 (t, 2H, −CH_2−CH_2−C=O(CH_3)), 2.12 (m, 3H, −CH_2−CH_2−C=O(CH_3)), 2.04 (m, 4aH, −CH_2−C(CH_3)=CH− CH_2−), 1.68 (m, 3aH, −CH_2−C(CH_3)=CH−CH_2−). FT-IR (ν, cm^{-1}): 1720 (-C=O); M_n (GPC) = 7.0 kDa, *Đ* = 3.2.

The oxidative degradation of SBR (SBRox): Yield = 72%. ^{1}H NMR (500 MHz, Chloroform-*d*) δ 9.76 (t, 1H, −CH_2−C*H*O), 7.2-6.91 (m, 5bH, -CH_2−CHCH_2−*Ph*), 5.71-4.68 (m, 2aH, −CH_2−C*H*=C*H*−CH_2−), 2.65-2.49 (m, bH, -CH_2−C*H*CH_2−Ph), 2.40 (t, *J* = 7.3 Hz, 2H, −CH_2−CHO), 2.28-1.66 (m, 4aH, −CH_2−CH=CH−CH_2−), 1.59-1.51 (m, bH, -CH_2−CHCH_2−Ph). FT-IR (ν, cm^{-1}): 1727 (-C=O); M_n (GPC) = 8.8 kDa, *Đ* = 1.8.

The oxidative degradation of NBR (NBRox): Yield = 78%. ^{1}H NMR (500 MHz, Chloroform-*d*) δ 9.76 (t, 1H, −CH_2−C*H*O), 5.68-5.35 (m, 2aH, −CH_2−C*H*=C*H*−CH_2−), 2.70-2.51 (t, bH, -CH_2−C*H*CH_2−CN), 2.46-2.42 (m, 2H, −CH_2−CHO), 2.44-1.93 (m, 4aH, −CH_2−CH=CH−CH_2−), 1.76-1.45 (m, 2bH, CH_2−CHCH_2−CN). FT-IR (v, cm^{-1}): 1728 (-C=O); M_n (GPC) = 16.9 kDa, *Đ* = 1.4.

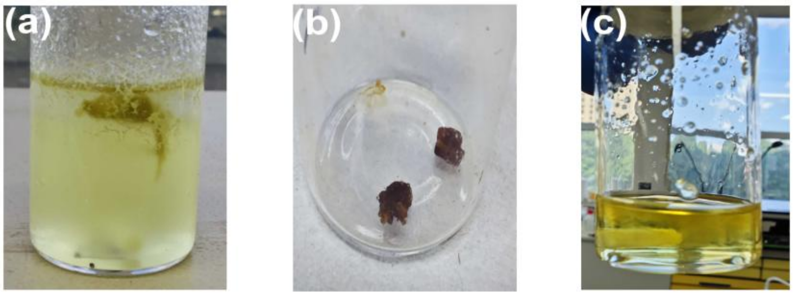

Figure S2. (a) Photographs of photoexcited oxidation of PBD by F2NB, some obvious insoluble substance suspended in the system. (b) The brown insoluble gel was separated after the F2NB oxidation of PBD; (c) Some insoluble mixture existed in the system of PBD oxidation using NBCN as oxidative.

2. 3. Photoexcited oxidative degradation of waste vulcanized unsaturated polymers

A waste nitrile glove (3.66 g) was cut into small pieces and was dispersed into 300 mL DCM. 2FNB (10.5 mL) and HFIP (7 mL) were added into the flask. The dispersion was placed under 390 nm household LED for oxidated degradation. After irradiation and stirring for 48 h, 100 mL cocktail solution (72 mL MeCN + 24 mL H_2O + 4 mL CH_2O + 1.84 g N-phenylmaleimide) was injected into the flask and fiercely stirring for 6 h to decompose oxidative intermediate. The mixture was centrifugated for 15 min and filtered to remove insoluble fraction (mainly fillers and pigments). The solution was concentrated to 40 mL by rotary evaporation. Then, the concentrated solution was precipitated by 200 mL cold methanol. The obtained precipitates were collected and dried in vacuum. A viscous gel was obtained by 63% yield for NMR analysis.

^{1}H NMR (500 MHz, Chloroform-*d*) δ 9.74 (m, 1H, −CH_2−C*H*O), 5.53-5.40 (m, 2aH, −CH_2−C*H*=C*H*−CH_2−), 2.72-2.43 (m, cH, -CH_2−C*H*CH_2−CN), 2.46-2.42 (m, 2H, −CH_2−CHO),

2.44-2.10 (m, 4aH, −CH_2−CH=CH−CH_2−), 2.06-1.95 (m, bH, -CH_2−$CH$$CH_2$−$S_x$−), 1.76-1.60 (m, 2cH, CH_2−CHCH_2−CN), 1.58-1.42 (m, 2bH, CH_2−CHCH_2−S_x−). FT-IR (v, cm^{-1}): 1727 (-C=O); M_n (GPC) = 17.5 kDa.

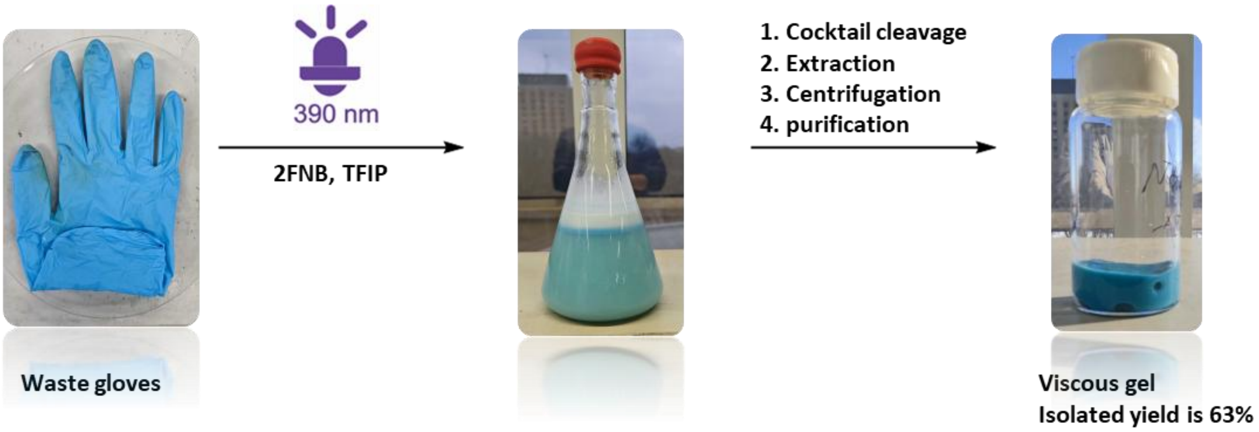

Figure S3. Photographs of photoexcited oxidation of waste gloves by 2FNB.

2.4 Construct network by dynamic imine chemistry

The methods of preparation elastomers are shown in Figure 3a. A series of elastomers was prepared following the same procedure. Cross-linking was achieved by mixing NBR gloves oxidative products (NBR GLox) and 1,4-phenylenediamine (PH2NH). 0.5 g of NBR GLox was dissolved in 5 mL $CHCl_3$/DMF = 1:1 (v/v). A varying mass ratio of PH2NH to NBR Glox was added into the flask under stirring for 2 h until they dispersed and reacted completely. The mixture was injected into the Teflon model as a container for heat cross-linking. The solvent evaporated, and the polymer was thermally cured at 60 °C for 16 h, and the final films were placed in vacuum at 80 °C for 4 h to obtain dynamic imine networks.

2.5 Degradation Study of regenerated rubber in Aqueous Media.

The hydrolytic degradation of N2-NBR GLox in 0.1 M NaOH, 0.1 M HCl and DI H_2O was examined under room temperature according to the following procedures. Each sample (about 30 mg) of N2-NBR GLox was immersed in the three different aqueous solutions (0.1 M NaOH, 0.1 M HCl and DI H_2O, each 3 mL) under room temperature. After appointed time, the samples were removed from the corresponding solutions. After dried in oven at 105 °C overnight, the samples were weighed. Then, the specimens were immersed again in the corresponding solutions, and the above-mentioned process was repeated at planned time. The results were shown in Figure S19.

2.6 Reprocessing of network by hot-pressing

A hot-pressing machine (Carver 20500-293) was employed to reprocess the recycled materials. The virgin films were cut into small pieces and placed into a steel mold between two steel sheets. For the reprocessing of N2-NBR GLox, it was heat pressed under a pressure of 10 MPa at 140 °C for 60 minutes to obtain new films.

2.7 Further oxidation from aldehyde of 2AD into carboxylic acid

In a 20 mL vial with magnetic stirrer, 2AD (540 mg, aldehyde content of 0.36mmol) was dissolved in 14.4 mL mixed solvent THF/DMF = 1:1 (v/v). Subsequently, the oxone (442 mg, 0.72 mmol) was added to the vials. The solution was fiercely stirring for 12 h at room temperature. After stirring, the mixture was filtered and washed with DCM (25 mL) and saturated NaCl solution (2×20 mL). The aqueous phase was extracted with DCM. The organic phases were combined and dried over moderate anhydrous Na_2SO_4. Afterwards, the solution was concentrated and purified by precipitation in methanol to obtain 2AD-COOH as a viscous liquid with a yield of 82%. ^{1}H NMR (500 MHz, Chloroform-*d*) 5.50-5.24 (m, 2aH, −CH_2−C*H*=C*H*−CH_2−), 2.38 (t, 4H, −CH_2−COOH), 2.18-1.96 (m, 4aH, −CH_2−CH=CH−CH_2−). FT-IR (v, cm^{-1}): 1713 (-COOH); M_n (GPC) = 8.1 kDa, M_w = 12.4 kDa, $Đ$ = 1.65.

2.8 Reduction of from aldehyde of 2AD into alcohol

In a 50 mL single round bottom flask with magnetic stirrer, 2AD (540 mg, aldehyde content of 0.36 mmol) was dissolved in 18 mL THF. Subsequently, $NaBH_4$ (41 mg, 1.08 mmol) was added to the vials. The solution was placed at room temperature stirring for 12h. After stirring, 5 mL methanol was slowly added into the round flask to quench the reductive. The mixture was filtered and washed with DCM (25 mL) and saturated NaCl solution (2×20 mL). The aqueous phase was extracted with DCM. The organic phases were combined and dried over moderate anhydrous Na_2SO_4. Afterwards, the solution was concentrated and purified by precipitation in methanol to obtain 2AD-OH as a viscous liquid with high yield of 85%. ^{1}H NMR (500 MHz, Chloroform-*d*) 5.46-5.33 (m, 2aH, $-CH_2-CH=CH-CH_2-$), 3.65 (t, 4H, $-CH_2-OH$), 2.28 (m, 4H, $-CH_2-CH_2-OH$), 2.16-2.01 (m, 4aH, $-CH_2-CH=CH-CH_2-$), 1.62 (t, 2H, $-CH_2-OH$); M_n (GPC) = 6.8 kDa, M_w = 10.1 kDa, $Đ$ = 1.49.

Table S1. Results of photoexcited oxidation of TFO by different nitroarenes (NAs) [a]

run	Nitroarenes (NAs)	Additives (add.)	TFO/NAs/Add. [b]	Time (h)	Temp (˚C)	Conv.[c] (%)	Yield[d] (%)
1	NB	2,6-Lutidine	1/3/1	12	0	28.6	13.8
2	NB	2,6-Lutidine	1/3/1	12	r.t.	26.0	16.5
3	NB	HFIP	1/3/2	12	r.t.	14.7	6.9
4	NBCN	-	1/1.5/0	12	r.t.	30.9	11.2
5	NBCN	-	1/3/0	12	r.t.	37.9	8.7
6	NBCN[d]	-	1/1.5/0	12	r.t.	31.1	16.6
7	F2NB	-	1/1.5/0	12	r.t.	39.4	32.0
8	F2NB	2,6-Lutidine	1/1.5/2	12	r.t.	25.3	17.9
9	F2NB	HFIP	1/1.5/2	12	r.t.	38.0	21.5
10	2FNB	-	1/1.5/0	12	r.t.	38.8	27.0
11	2FNB	-	1/1.5/0	24	r.t.	40.8	33.7
12	2FNB	-	1/3/0	12	r.t.	46.7	32.4
13	2FNB	2,6-Lutidine	1/1.5/1	24	r.t.	29.3	27.1
14	2FNB	2,6-Lutidine	1/1.5/2	24	r.t.	28.5	27.9
15	2FNB	HFIP	1/1.5/2	24	r.t.	50.7	25.7
16	2FNB	HFIP	1/3/4	12	r.t.	43.9	36.7

[a]Oxidations were performed under 390 nm LED irradiation, using different nitroarenes as photo-excited oxidatives, using DCM as solvent at room temperature for 12h. The concentration of TFO is 0.33 M. [b]The NAs and Add. equivalent was calculated relative to the molar amount of TFO. [c]As calculated through ^{1}H NMR spectra. [d]Yield of aldehyde as calculated through ^{1}H NMR spectra. [d]Using the same concentration of MeCN as solvent. (NB = nitrobenzene; NBCN = 4-nitrobenzonitrile; F2NB = 1,3-dinitro-5-(trifluoromethyl)benzene; 2FNB = 1-Nitro-3,5-bis(trifluoromethyl)benzene; HFIP = 1,1,1,3,3,3-Hexafluoropropan-2-ol).

Table S2. Results of photoexcited oxidation of TFO by using different solvent [a]

run	TFO/2FNB/TFIP[b]	Solvent	Concentration (M)	Time (h)	Temp. (˚C)	Conv.[c] (%)	Yield[d] (%)
1	1/1.5/0	-	-	12	r.t.	28.0	20.3
2	1/1.5/0	DCM	0.33	12	r.t.	38.8	27.0
3	1/1.5/0	MeCN	0.33	12	r.t.	29.9	21.5
4	1/1.5/0	EtOAc	0.33	12	r.t.	26.9	17.6
5	1/1.5/0	Acetone	0.33	12	r.t.	25.9	20.5
6	1/1.5/0	THF	0.33	12	r.t.	32.8	15.9
7	1/1.5/0	$CHCl_3$	0.33	12	r.t.	34.5	24.1

[a]Oxidations were performed by using different solvent at room temperature for 12h under 390 nm LED irradiation, using 2FNB as photo-excited oxidatives. [b]The NAs and Add. equivalent was calculated relative to the molar amount of TFO. [c]As calculated through ^{1}H NMR spectra. [d]Yield of aldehyde as calculated through ^{1}H NMR spectra.

Table S3. Results of photoexcited oxidation of TFO under different wavelength LED irradiation [a]

run	Nitroarenes (NAs)	TFO/NAs/Add. [b]	Wavelength (nm)	Time (h)	Temp (˚C)	Conv.[c] (%)	Yield[d] (%)
1	2FNB	1/1.5/0	365	12	r.t.	35.0	15.9
2	2FNB	1/1.5/0	390	12	r.t.	38.8	27.0
3	2FNB	1/1.5/0	470	12	r.t.	11.9	7.7
4	NBCN	1/1.5/0	365	12	r.t.	41.7	26.7
5	NBCN	1/1.5/0	390	12	r.t.	30.9	11.2

[a]Oxidations were performed under different wavelength LED irradiation, using different nitroarenes as photo-excited oxidatives, using DCM as solvent at room temperature for 12h. The concentration of TFO is 0.33 M. [b]The NAs and Add. equivalent was calculated relative to the molar amount of TFO. [c]As calculated through ^{1}H NMR spectra. [d]Yield of aldehyde as calculated through ^{1}H NMR spectra.

Table S4. Optimized results of photoexcited oxidation of PBD [a]

run	PBD (mmol)	PBD/2FNB/TFIP[b]	Concentration (mM)	Time (h)	Temp (˚C)	Conv.[c] (%)	Yield[d] (%)	Yield[e] (%)
1	0.8	1/1/2	80	12	r.t.	21.5	8.5	84
2	0.8	1/2/2	80	12	r.t.	23.3	11.9	72
3	0.8	1/3/2	80	12	r.t.	27.8	14.1	82
4	0.8	1/3/4	80	12	r.t.	28.5	15.6	76
5	0.8	1/3/2	80	12	r.t.	30.0	19.3	83
6	0.8	1/3/4	20	12	r.t.	28.1	15.2	78
7	0.8	1/3/4	40	12	r.t.	33.7	16.7	86
8	80.0	1/1/1	80	16	r.t.	23.2	13.8	82

[a]Oxidations were performed under 390 nm LED irradiation, using 2FNB as photo-excited oxidatives, using DCM as solvent at room temperature for 12h. The concentration of PBD is 80 mM. [b]The 2FNB and HFIP equivalent was calculated relative to the molar amount of olefinic bonds in the polymer. [c]As calculated through ^{1}H NMR spectra. [d]Yield of aldehyde as calculated through ^{1}H NMR spectra. [e]isolated yield of 2AD. [f]Partly insoluble products precipitated.

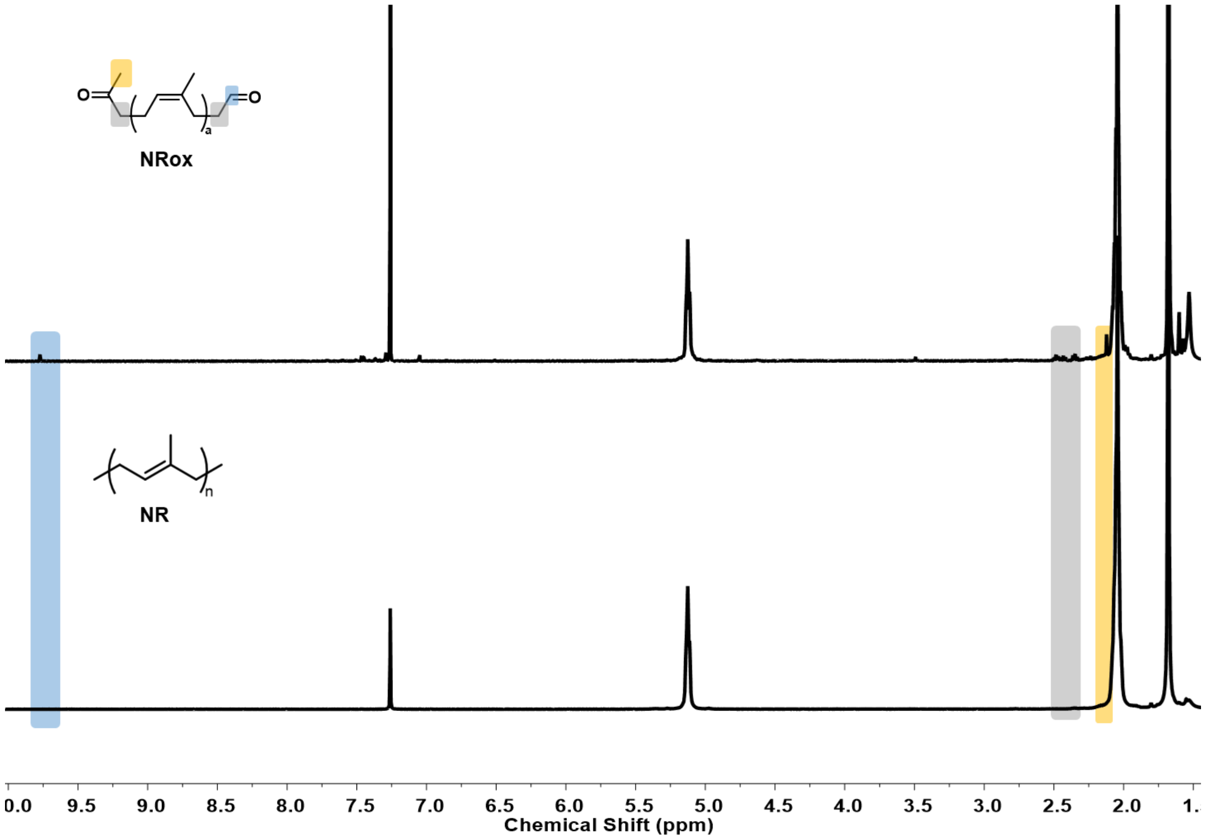

Figure S4. ^{1}H NMR spectrum of NR and NRox after purification by MeOH

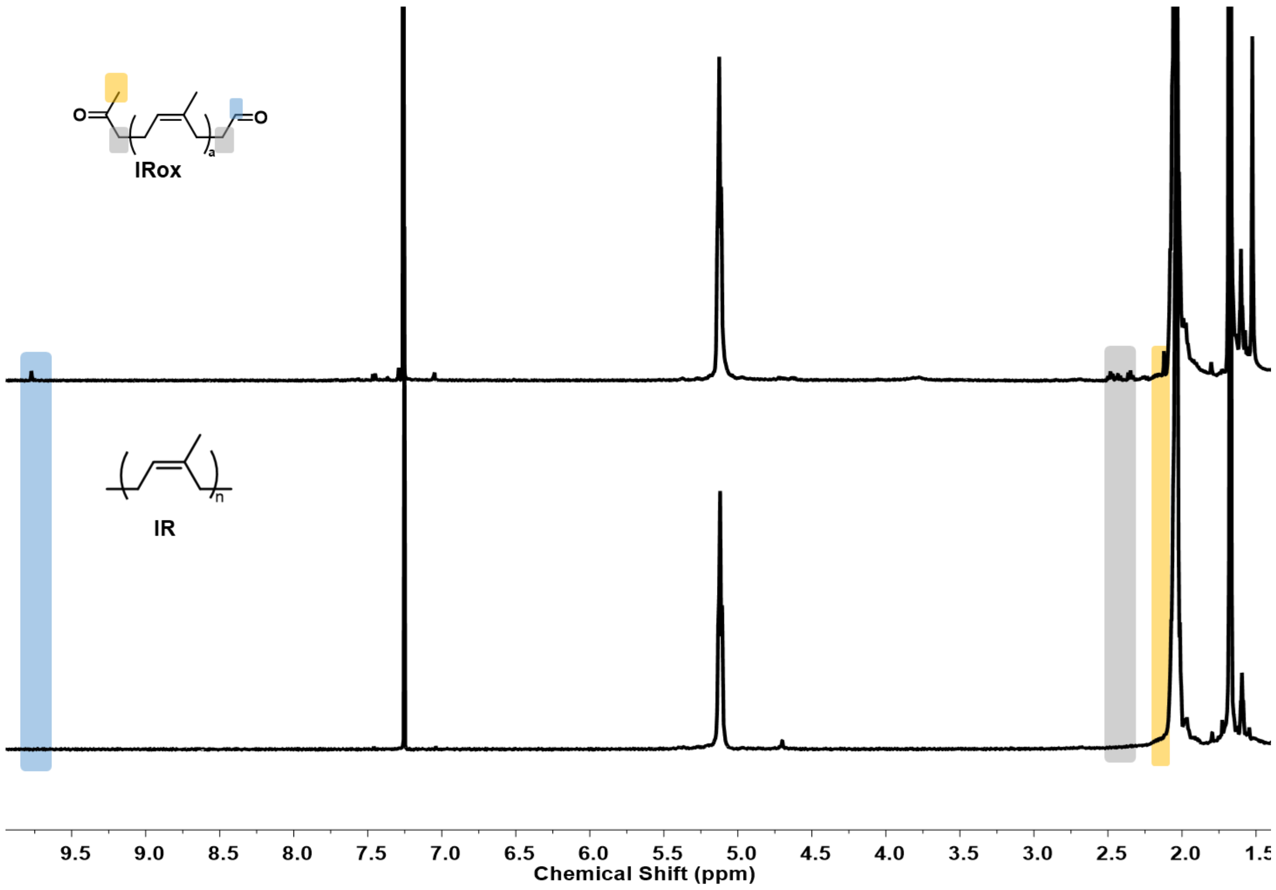

Figure S5. ^{1}H NMR spectrum of IR and IRox after purification by MeOH

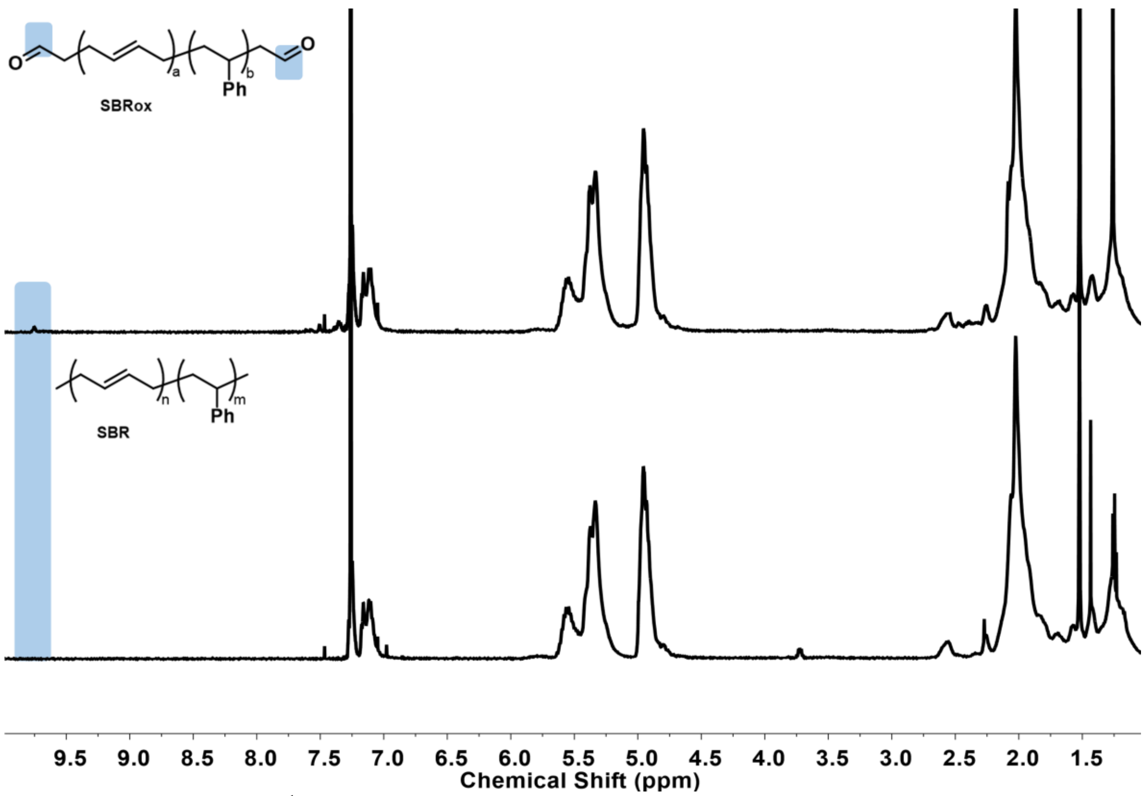

Figure S6. ^{1}H NMR spectrum of SBR and SBRox after purification by MeOH

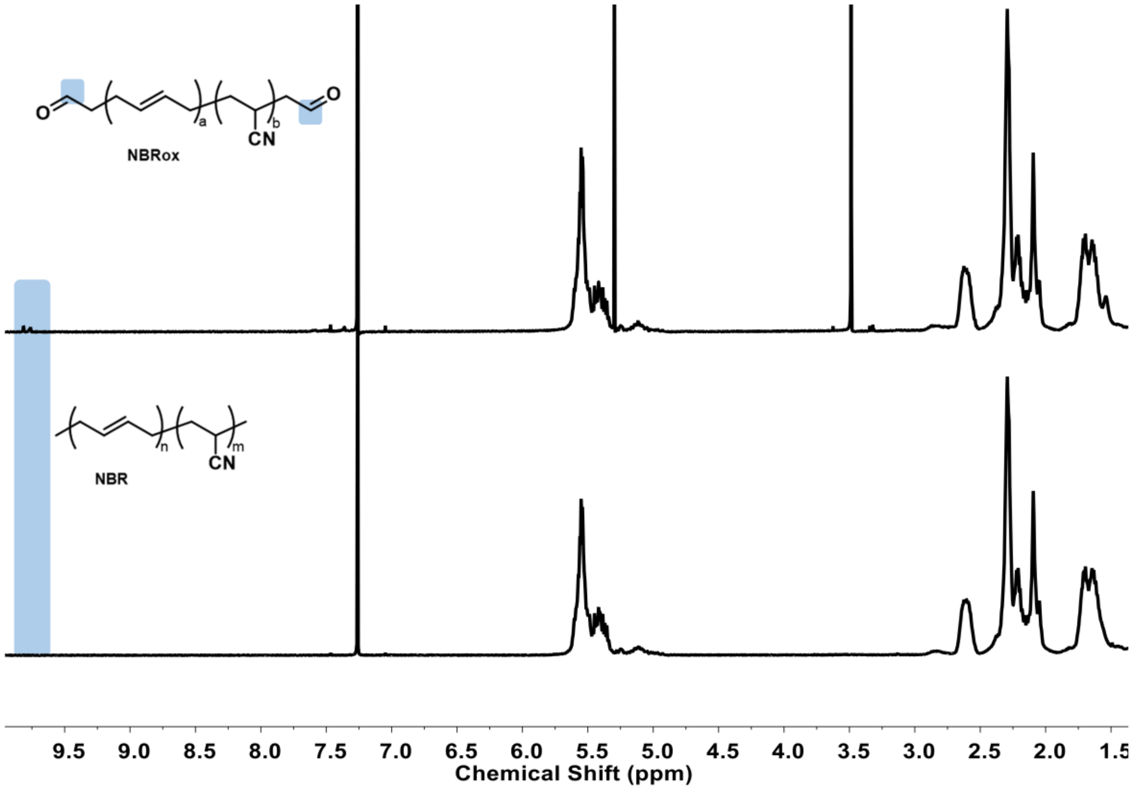

Figure S7. ^{1}H NMR spectrum of NBR and NBRox after purification by MeOH

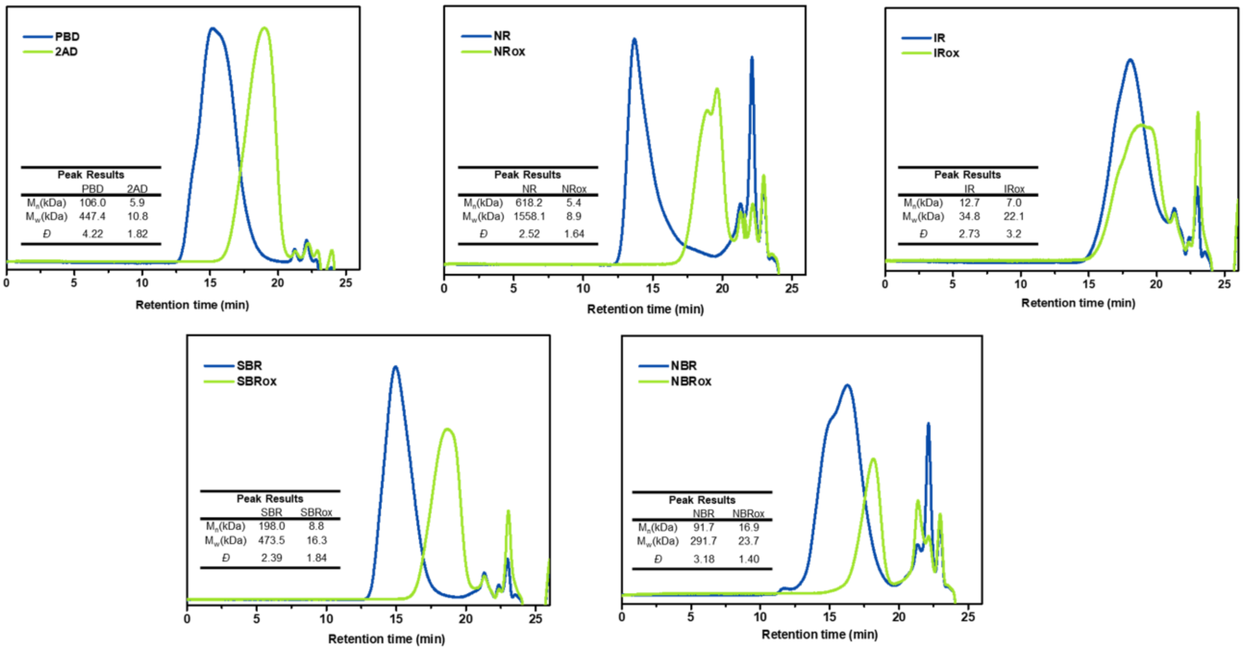

Figure S8. GPC traces of different unsaturated polymers and photo-oxidation products

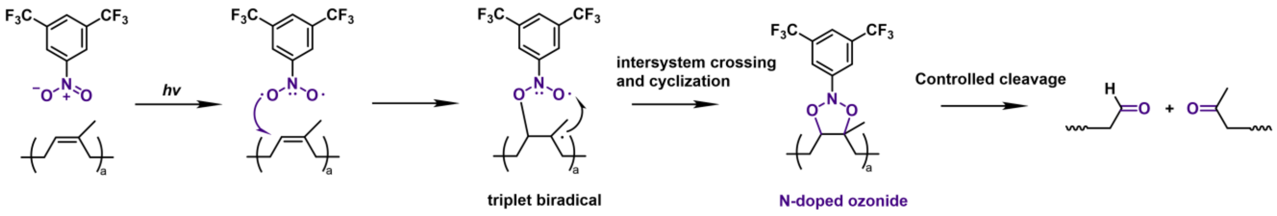

Figure S9. Proposed mechanism of NR photochemical degradation

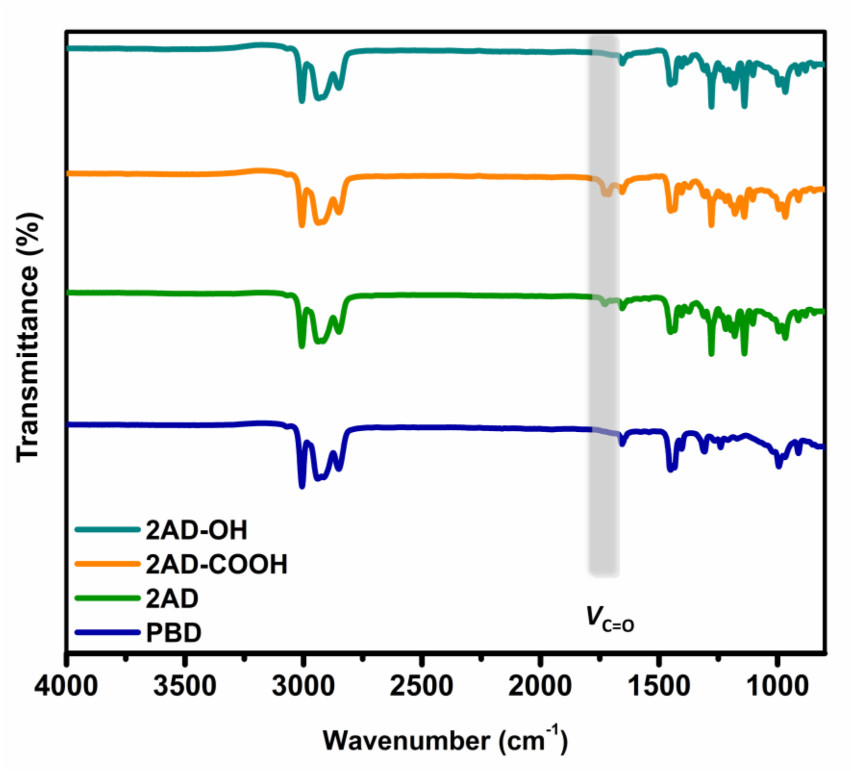

Figure S10. FT-IR spectra of PBD and 2AD and 2AD-OH and 2AD-COOH

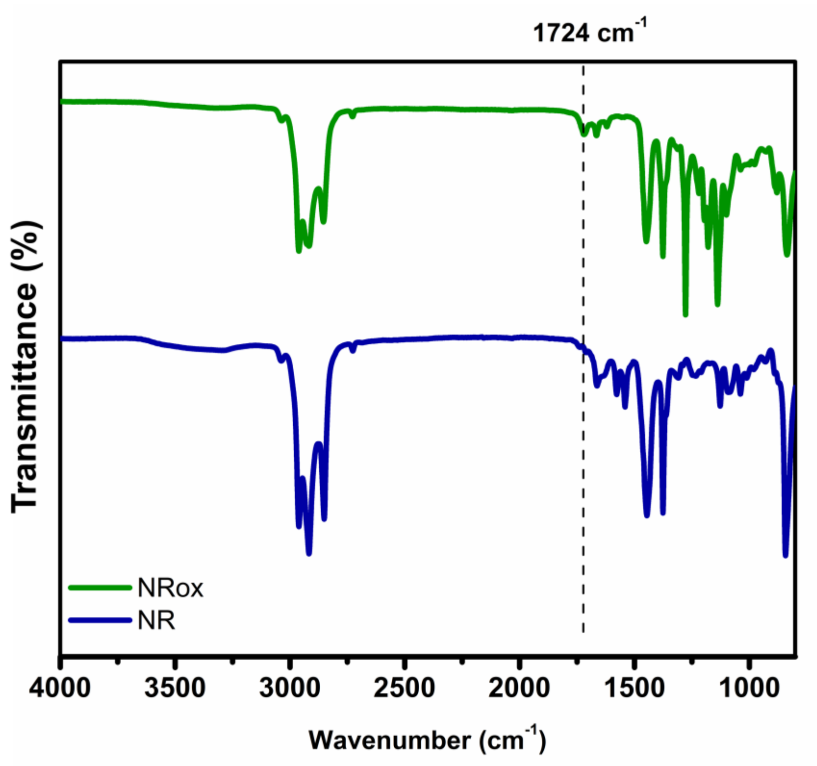

Figure S11. FT-IR spectra of NR and NRox

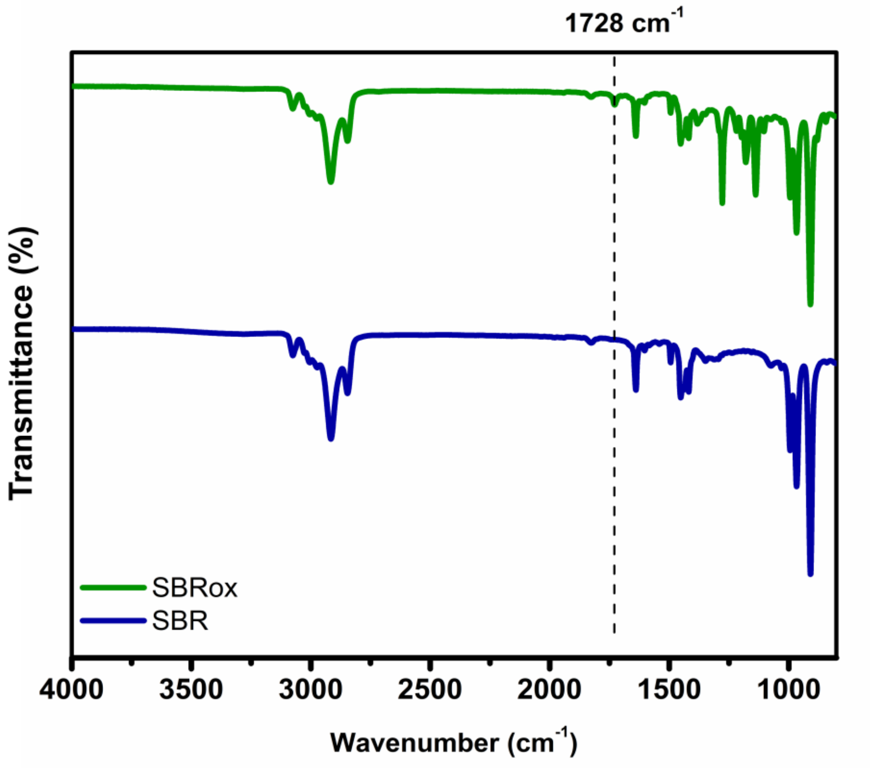

Figure S12. FT-IR spectra of SBR and SBRox

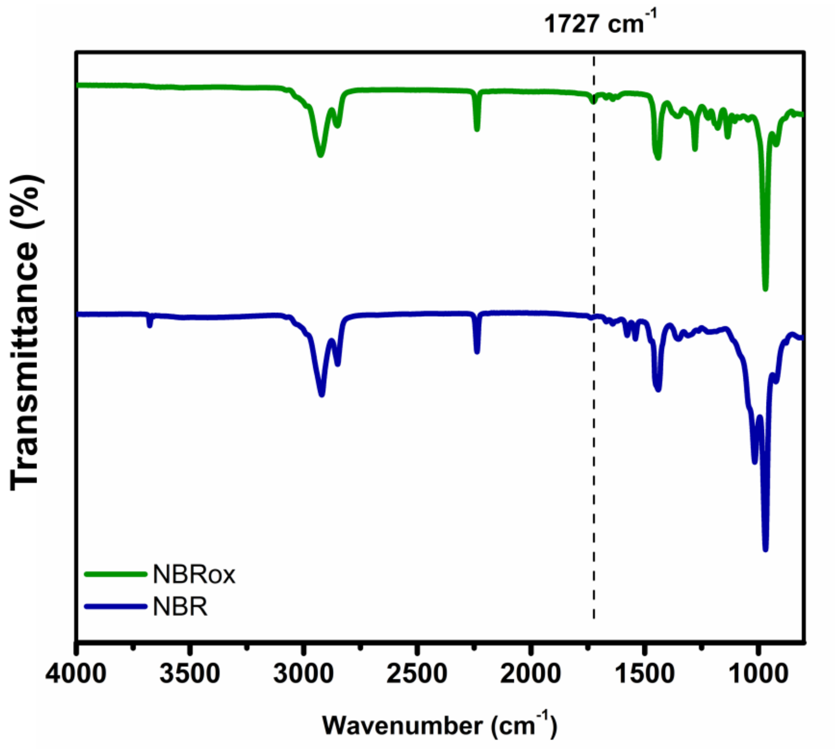

Figure S13. FT-IR spectra of NBR and NBRox

Table S5. Kinetics results of NR photo-oxidation

		Kinetics Results		
		Yield (%)	Mn (kDa)	*Đ*
	0 min	0	57.0	3.54
	30 min	5.6	45.2	2.77
Light off 30min	60 min	6.0	28.4	2.09
	90 min	6.1	28.8	2.31
Light off 60min	120 min	6.5	19.1	2.08
	180 min	6.5	20.0	2.12
	300 min	9.3	8.4	1.81
	420 min	11.4	6.6	1.72
	540 min	13.2	4.3	1.56
	660 min	14.4	2.4	2.01

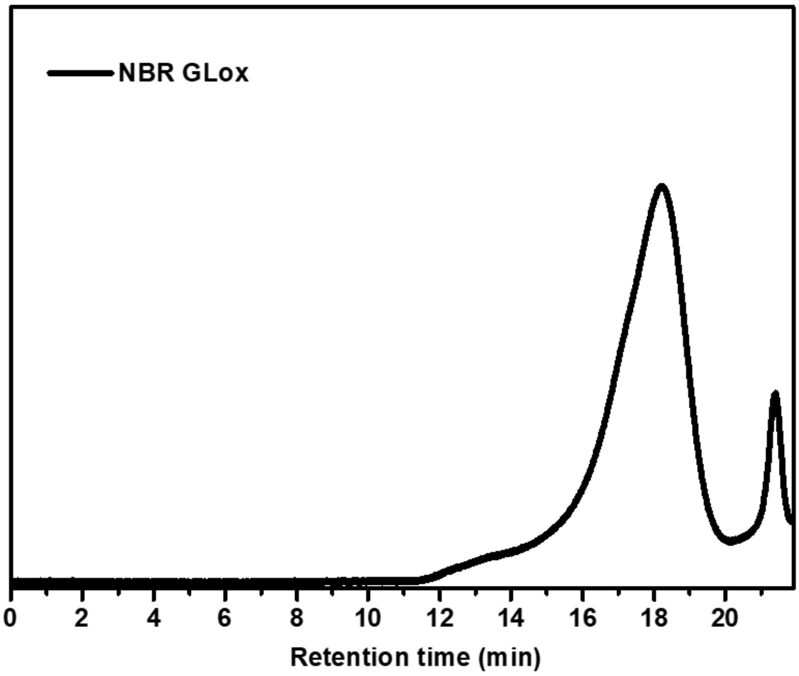

Figure S14. GPC traces of NBR GLox

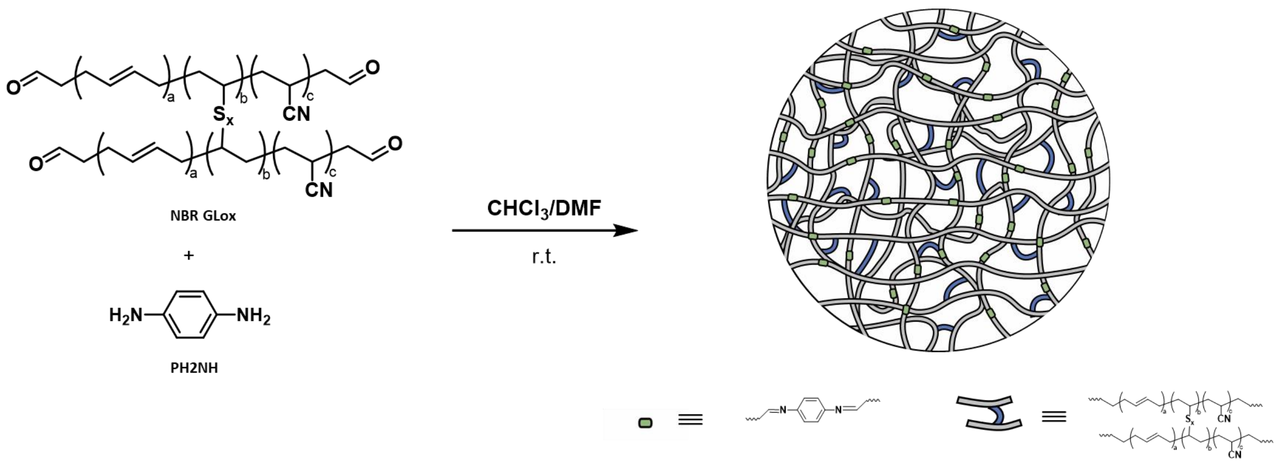

Figure S15. Scheme of construction network form NBR Glox with PH2NH

Table S6. Optimized results of different ratio of NBR GLox and PH2NH to construct networks

	NBR GLox（mg）	PH2NH (mg)	PH2NH (wt%)	Tensile strength (MPa)	Strain (%)
N1-NBR GLox	250	2	0.8	8.2	234
N2-NBR GLox	250	5	2.0	15.4	282
N3-NBR GLox	250	10	3.9	7.9	110
N4-NBR GLox	250	25	9.1	7.5	117

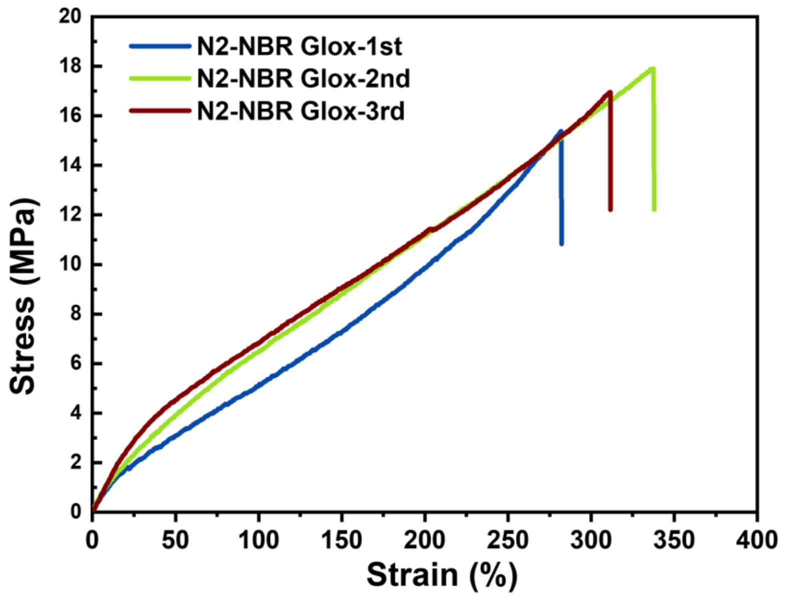

Figure S16. Stress-strain curves of N2-NBR GLox (×3 samples each)

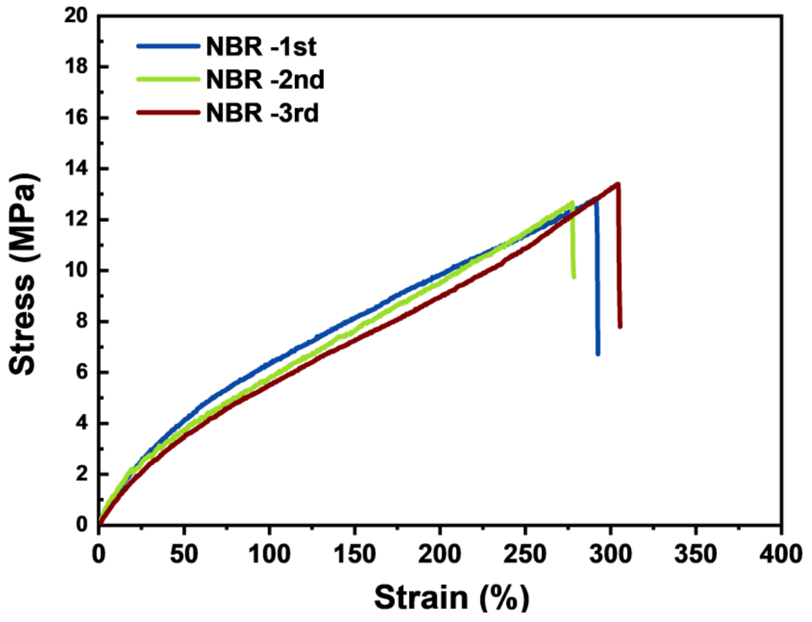

Figure S17. Stress-strain curves of commercial NBR (×3 samples each)

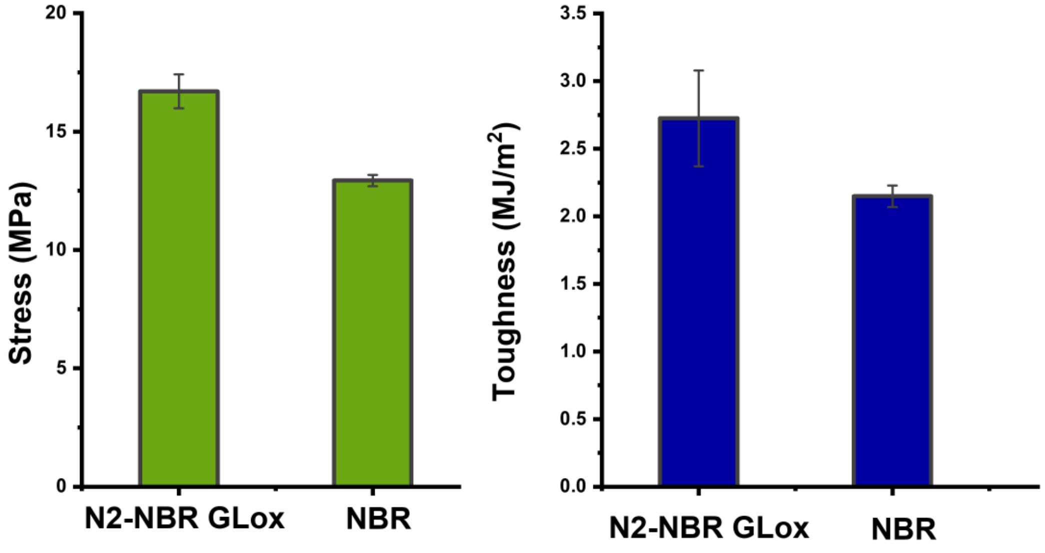

Figure S18. Mechanical properties of stress and toughness

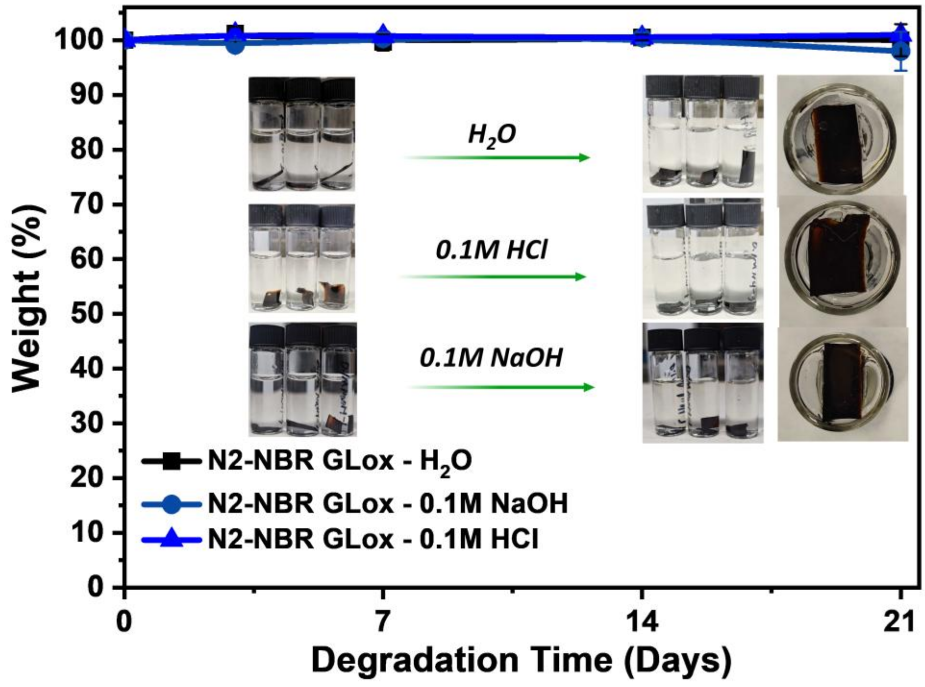

Figure S19. Degradation profiles of N2-NBR GLox at room temperature in DI water, 0.1M NaOH (aqueous), and 0.1M HCl (aqueous).

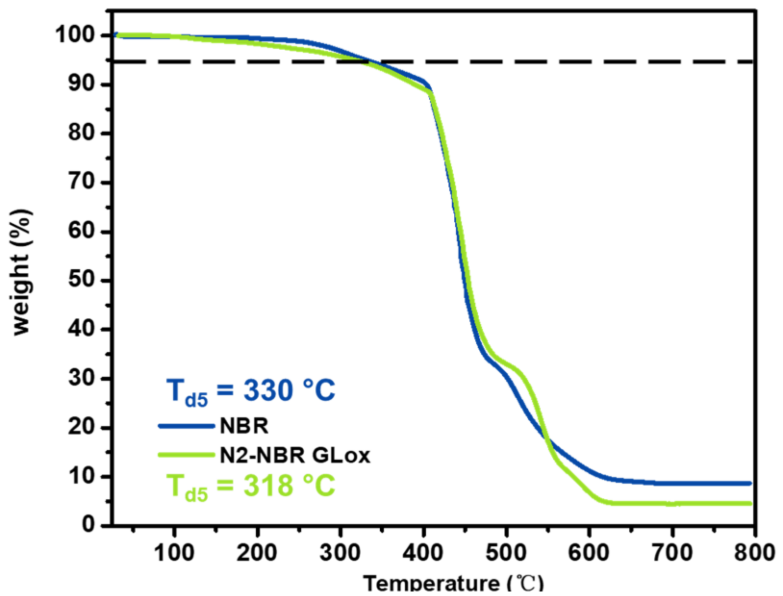

Figure S20. TGA curves of NBR and N2-NBRGLox in nitrogen

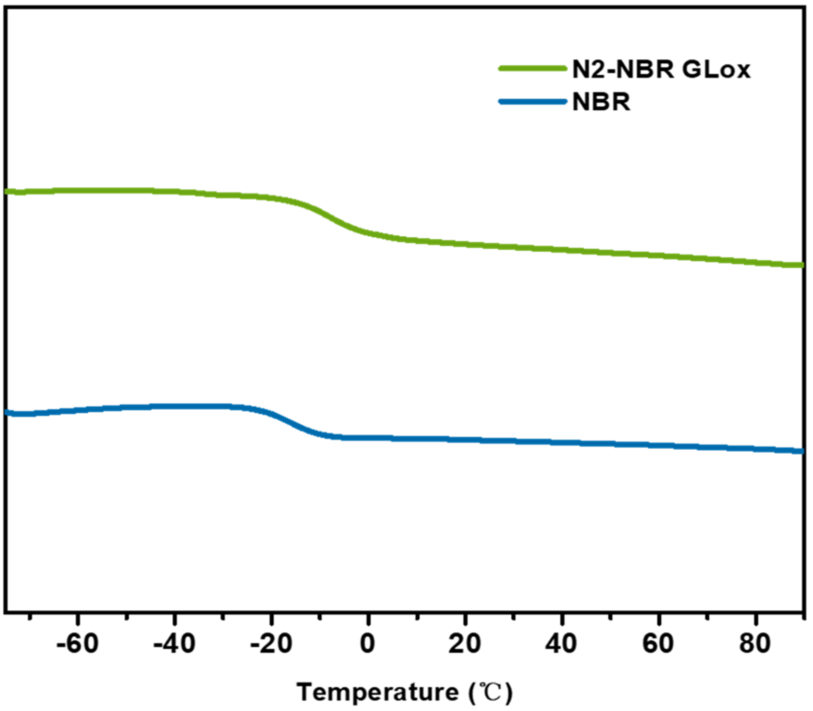

Figure S21. DSC traces of NBR and N2-NBRGLox in nitrogen

Table S7. Optimized results of different ratio of further oxidation from 2AD into 2AD-COOH[a]

run	2AD/mmol	oxone/mmol	2AD/oxone	Solvent [b]	Concentration (mM)	Time (h)	Conv.[c] (%)	Yield[d] (%)
1	0.036	0.072	1/2	DMF/H_2O=1/1	50	3	15	n.d.
2	0.036	0.072	1/2	THF/H_2O=1/1	25	3	24	n.d.
3	0.036	0.036	1/1	DMF/THF=1/1	50	3	79	78
4	0.036	0.072	1/2	DMF/THF=1/1	25	3	91	84
5	0.360	0.720	1/2	DMF/THF=1/1	25	12	93	82

[a]Further oxidations were performed by using oxone as oxidatives, DMF/THF as cosolvent for 12h at room temperature. The concentration of 2AD is 25 mM. [b]It is the volume ratio. [c]As calculated through ^1H NMR spectra. [d]isolated yield of 2AD-COOH.

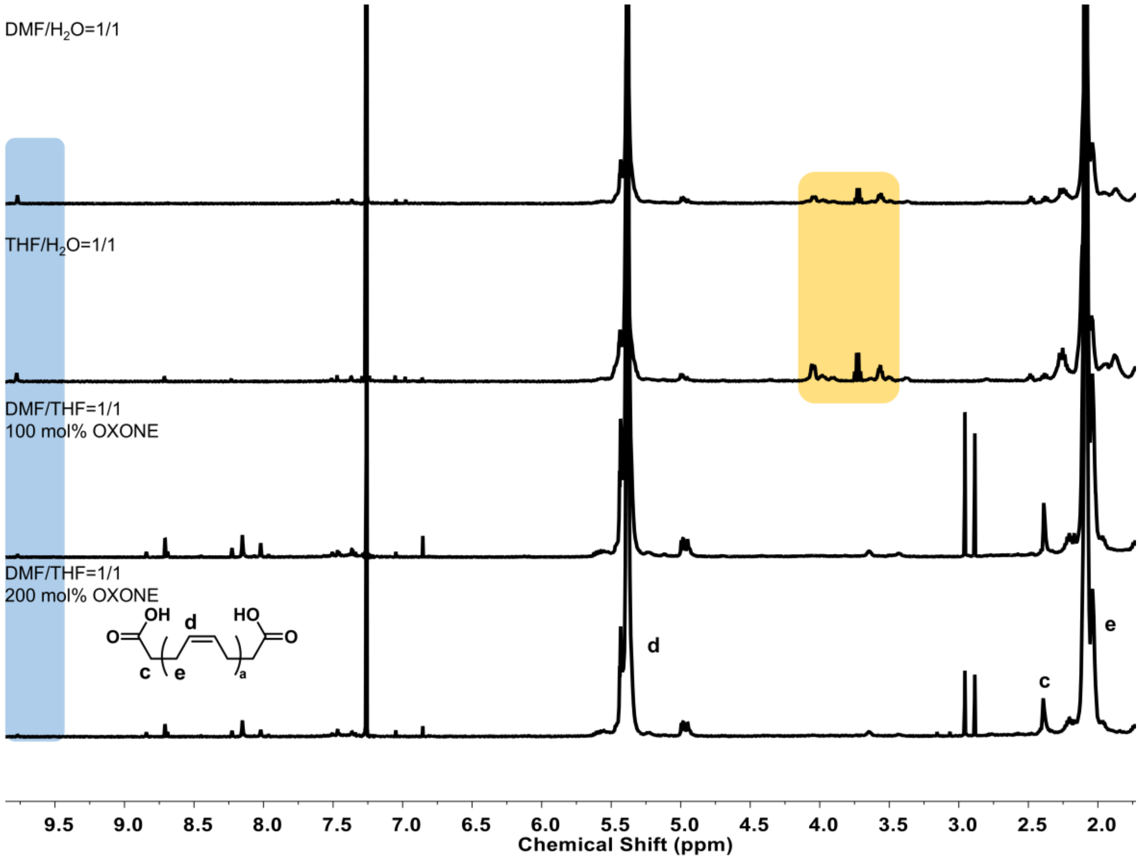

Figure S22. ^1H NMR spectrum of 2AD-COOH using different solvents

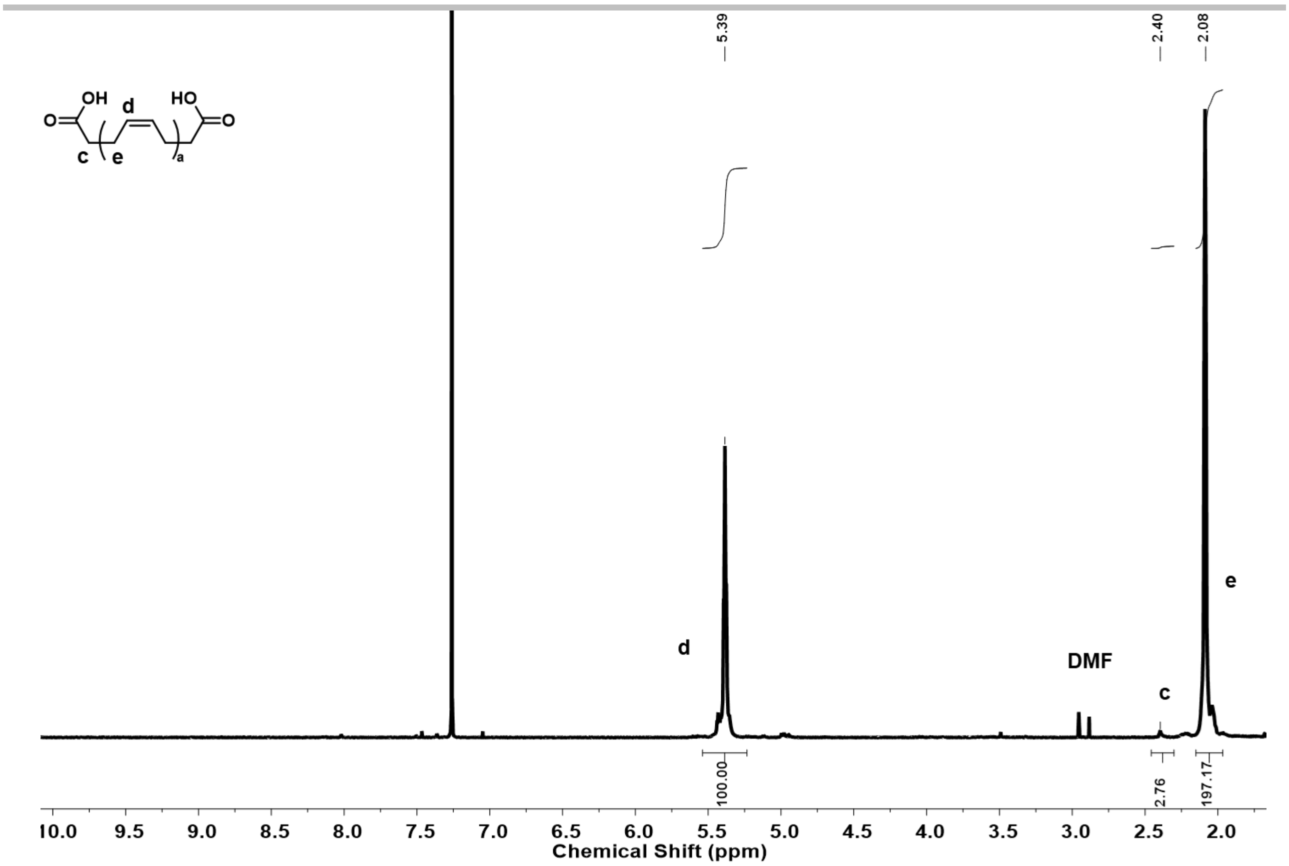

Figure S23. ^{1}H NMR spectrum of 2AD-COOH

Table S8. Optimized results of reduction of 2AD prepolymer to obtain 2AD-OH [a]

run	2AD/mmol	$NaBH_4$/mmol	2AD/$NaBH_4$	Solvent	Concentration (mM)	Time (h)	Conv.[b] (%)	Yield[c] (%)
1	0.036	0.22	1/6	THF	20	12	100	82
2	0.036	0.44	1/12	THF	20	12	100	73
3	0.360	1.08	1/3	THF	40	12	100	85

[a]Reduction of 2AD was performed by using $NaBH_4$ as reductive, THF as solvent for 12h at room temperature. The concentration of 2AD is 40 mM. [b]As calculated through ^{1}H NMR spectra. [c]isolated yield of 2AD-COOH.

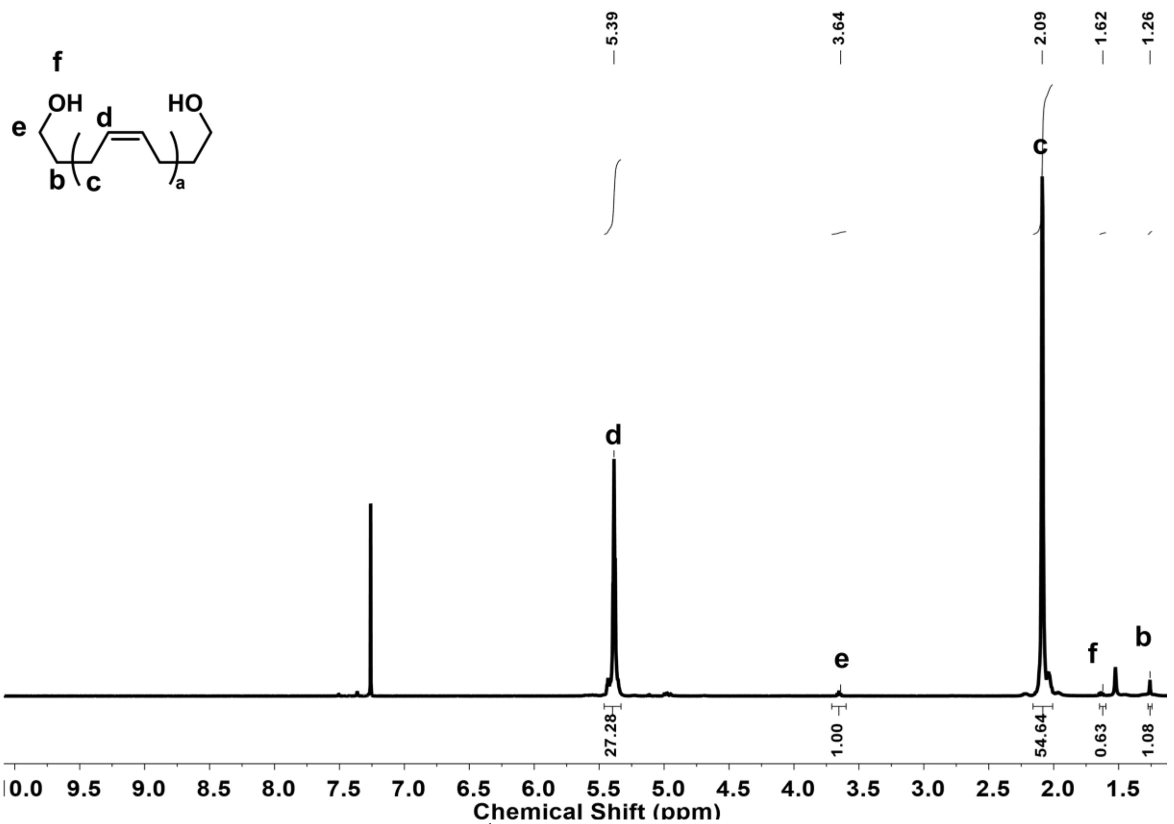

Figure S24. ^{1}H NMR spectrum of 2AD-OH

References:

[1] H. Chen, X. Guan, P. Zhang, D. Sathe, J. Wang, *Cell Reports Physical Science* **2024**, *5*, 102104.
